# Retinal Vasculature as a Non-Invasive Window for Preeclampsia Prediction and Assessment: From Fundoscopy to Artificial Intelligence-Assisted Retinal Imaging

**DOI:** 10.3390/diagnostics16213676

**Published:** 2026-11-06

**Authors:** Dimitris Baroutis, Maria Fanaki, Konstantinos Koukoumpanis, Ioannis Fragiskos, Nikolaos Sindos, Michael Sindos, Marianna Theodora, Vasilios Pergialiotis, George Daskalakis

**Affiliations:** 1st Department of Obstetrics and Gynecology, Alexandra Hospital, National and Kapodistrian University of Athens, 11528 Athens, Greecegdaskalakis@yahoo.com (G.D.)

**Keywords:** preeclampsia, retinal imaging, retinal vessel calibre, optical coherence tomography angiography, deep learning, artificial intelligence, screening, endothelial dysfunction

## Abstract

Preeclampsia is a leading cause of maternal and perinatal morbidity and mortality, and effective prevention depends on the early identification of women at risk. Because the disorder is fundamentally a state of systemic endothelial dysfunction and microvascular maladaptation, the retina has long attracted interest as a candidate in vivo biomarker. It is the only vascular bed that can be visualised directly, repeatedly and non-invasively. This review appraises retinal assessment across four modality classes, classifying each study by the timing of imaging relative to diagnosis to distinguish early prediction from diagnostic discrimination, severity assessment and long-term follow-up. Qualitative fundoscopy reliably grades hypertensive retinopathy but is subjective and insensitive in mild disease. Quantitative retinal vessel analysis links narrower retinal vessels with the hypertensive disorders of pregnancy; in one longitudinal cohort, pressure-corrected arteriolar and venular calibre was reduced from 13 weeks in women who later developed preeclampsia, although effects were modest. Optical coherence tomography (OCT) and OCT angiography studies, almost all performed after diagnosis, report choroidal and microvascular changes of inconsistent direction and, therefore, inform diagnosis and severity assessment rather than prediction. Ophthalmic artery Doppler may offer adjunctive discrimination. Two single-centre deep learning models applied to early-pregnancy fundus photographs reported areas under the curve of 0.87–0.88 for preeclampsia, but without external validation, calibration or head-to-head comparison with established first-trimester screening. Retinal imaging is, therefore, a mechanistically grounded but unproven avenue for preeclampsia prediction. Prospective pre-diagnostic cohorts, standardised acquisition, adjustment for blood pressure and other confounders, and externally validated, calibrated models tested for incremental value over established screening are required. Only then can retinal biomarkers, particularly artificial intelligence-assisted analysis with portable fundus cameras, be adopted as a scalable screening adjunct, including in low-resource settings.

## 1. Introduction

Preeclampsia is a multisystem, pregnancy-specific disorder that complicates approximately 2–8% of pregnancies and accounts for a substantial proportion of maternal deaths, iatrogenic preterm birth and foetal growth restriction globally [1,2]. It is conventionally defined by new-onset hypertension after 20 weeks of gestation accompanied by proteinuria or evidence of maternal end-organ dysfunction. Contemporary classifications by the International Society for the Study of Hypertension in Pregnancy (ISSHP) and the American College of Obstetricians and Gynecologists (ACOG) have broadened the diagnostic criteria to encompass uteroplacental dysfunction even in the absence of proteinuria [3,4,5,6]. The syndrome is clinically heterogeneous, ranging from mild, late-onset disease to fulminant early-onset preeclampsia, eclampsia and the haemolysis, elevated liver enzymes and low platelets (HELLP) syndrome. This heterogeneity reflects a complex and incompletely understood pathophysiology [7,8].

The global burden of the disorder is profound and unequally distributed. Preeclampsia and the related hypertensive disorders contribute to an estimated tens of thousands of maternal deaths and a far greater number of perinatal deaths each year. The overwhelming majority occur in low- and middle-income countries, where access to antenatal surveillance and timely delivery is constrained [1,2,9,10]. Even in high-income settings, the disorder drives a substantial fraction of iatrogenic preterm births and neonatal intensive-care admissions. Contemporary classifications have moved away from a proteinuria-centred definition toward one that recognises the multisystem nature of the disease and the prognostic importance of maternal end-organ and uteroplacental dysfunction. They also increasingly distinguish early-onset (delivery before 34 weeks) from late-onset phenotypes, which differ in their degree of placental pathology, their maternal cardiovascular profile and, plausibly, their microvascular signature [3,4,5,11]. This phenotypic distinction is directly relevant to retinal biomarker research because a marker that performs well for early-onset, placentally driven disease may behave quite differently in the more common late-onset form.

The single most important advance in the management of preeclampsia over the past decade has been the demonstration that pharmacological prophylaxis is effective when initiated early in high-risk women. The Aspirin for Evidence-Based Preeclampsia Prevention (ASPRE) trial by Rolnik et al. [12,13] enrolled women identified as high-risk by the Fetal Medicine Foundation (FMF) first-trimester combined algorithm. In these women, low-dose aspirin commenced before 16 weeks reduced the incidence of preterm preeclampsia by approximately 60% [12,14]. This places a premium on accurate, early risk stratification. The FMF competing-risks model integrates maternal characteristics with mean arterial pressure, uterine artery pulsatility index and serum placental growth factor (PlGF). It identifies roughly three-quarters of women who will develop preterm preeclampsia at a 10% screen-positive rate and consistently outperforms risk factor checklists alone [15,16,17,18,19,20,21,22]. Nevertheless, the requirement for specialised ultrasound and biochemical assays limits the global scalability of such screening, particularly in low- and middle-income settings, where the burden of preeclampsia is greatest [23].

Against this background, the retinal microvasculature has re-emerged as an attractive target for non-invasive risk assessment. The retina is uniquely accessible: its arterioles and venules can be visualised, photographed and quantified directly, and its capillary networks can now be resolved layer-by-layer without contrast injection. Because preeclampsia is, at its core, a disorder of generalised endothelial activation and microvascular dysfunction, changes in the retinal circulation might plausibly mirror, and conceivably antedate, the systemic process [24,25,26]. The proposition that “the eye is a window” to systemic vascular health is well established in cardiovascular and cerebrovascular medicine [27,28]. The convergence of high-resolution imaging hardware with deep learning image analysis has reinvigorated interest in translating this principle to obstetrics.

Several reviews have addressed parts of this field. In narrative reviews, Soullane et al. [29] summarised the clinical ocular manifestations of preeclampsia, and Christou et al. [30] summarised the chorioretinal optical coherence tomography angiography (OCTA) literature, identifying seven OCTA studies through a single-database search. In a recent systematic review of retinal imaging in preeclamptic pregnancy, covering optical coherence tomography (OCT), OCTA, fundus photography and central retinal artery Doppler, Kitmiridou et al. [31] found the evidence inconclusive. They judged all 24 included studies to be at moderate or serious risk of bias. Meta-analyses by Sapantzoglou et al. [32] and Liu et al. [33] have examined, respectively, the added value of ophthalmic artery Doppler and the performance of machine learning models for preeclampsia prediction in general. To our knowledge, however, no previous synthesis has considered qualitative fundoscopy, quantitative vessel analysis, depth-resolved imaging and deep learning analysis of fundus photographs within a single pathophysiological framework. Nor has any previous synthesis classified each study according to the timing of retinal assessment relative to the diagnosis of preeclampsia, thereby separating evidence that bears on prediction from evidence that describes established disease. Finally, none have appraised the validation conditions of retinal prediction models against current prediction model standards and against the intended uses of the screening tools already in clinical practice.

This narrative review surveys the trajectory of retinal assessment in preeclampsia across four overlapping eras. These comprise (i) qualitative fundoscopy and hypertensive retinopathy grading; (ii) quantitative retinal vessel analysis (QRVA) of vessel calibre and geometry; (iii) depth-resolved imaging with OCT and OCTA; and (iv) artificial intelligence (AI)-assisted analysis of retinal fundus photographs. This review had four specific objectives. The first was to summarise the pathophysiological rationale linking the placental and systemic disturbances of preeclampsia to the retinal and choroidal circulations. The second was to appraise the evidence for each modality separately for early prediction, diagnostic discrimination, severity assessment and long-term follow-up, with ophthalmic artery Doppler considered as a complementary ocular vascular measure. The third was to evaluate the methodological quality of the key studies, their handling of confounders and, for prediction models, their development and validation conditions. The fourth was to define how retinal markers should be benchmarked against, and potentially integrated with, established screening tools, and what research is required before clinical adoption. A quantitative pooling of effect estimates was not an aim, given the heterogeneity of the literature.

**Figure 1:**
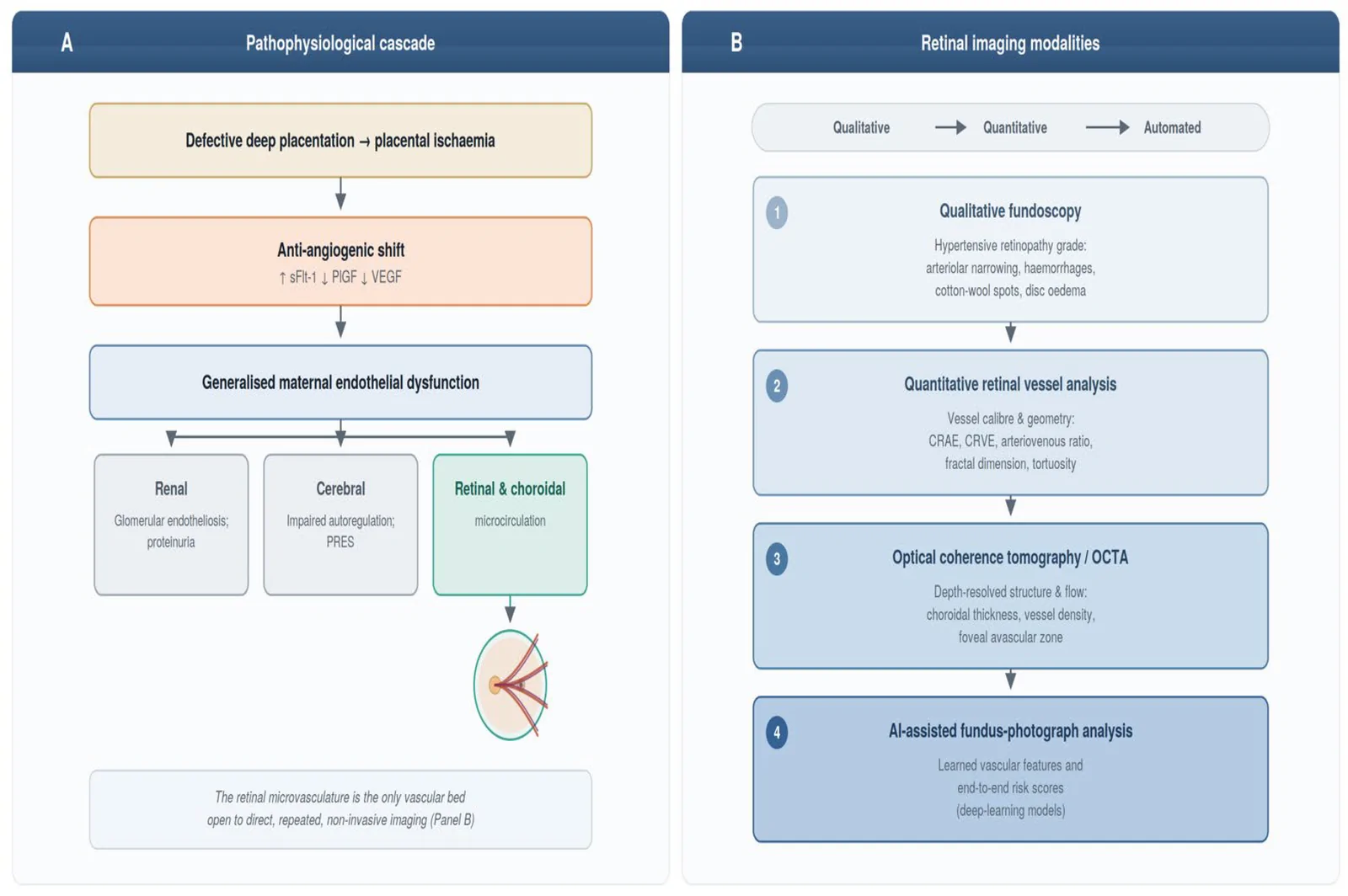
Conceptual overview of the placental–renal–retinal microvascular axis in preeclampsia. Panel (**A**): defective placentation and placental ischaemia drive an anti-angiogenic shift (↑sFlt-1; ↓PlGF/VEGF) and generalised endothelial dysfunction affecting the renal, cerebral and retinal/choroidal beds. Panel (**B**): the four retinal imaging modalities and their principal parameters, arranged from qualitative to automated assessment. PlGF, placental growth factor; PRES, posterior reversible encephalopathy syndrome; sFlt-1, soluble fms-like tyrosine kinase-1; VEGF, vascular endothelial growth factor.

Figure 1 summarises schematically the pathophysiological framework linking the placental, renal, cerebral and retinal microcirculations, together with the progression of imaging modalities used to interrogate the retinal bed. It provides the conceptual basis for the sections that follow.

A structured literature search was conducted, and this review was reported with reference to the PRISMA 2020 template and the SANRA criteria for narrative reviews [34,35]. The evidence base comprises 41 studies, including three identified after the database search [36,37,38]. The search strategy, study selection, evidence classification and quality appraisal [39,40,41,42,43] are detailed in the Supplementary Materials (Section S1, Table S1 and Figures S1 and S2). Throughout this review, studies are classified by the timing of retinal assessment relative to diagnosis as early prediction (Tier A), diagnostic discrimination (Tier B), severity assessment (Tier C) or long-term follow-up (Tier D).

## 2. Pathophysiological Rationale: The Placental–Renal–Retinal Microvascular Axis

Contemporary understanding frames preeclampsia as a two-stage disorder [44,45,46]. The first stage is defective deep placentation: incomplete trophoblastic remodelling of the maternal spiral arteries produces a high-resistance, intermittently hypoperfused placenta. The second, clinically manifest stage results from the release of placental factors into the maternal circulation that provoke a generalised maternal inflammatory and endothelial response [7,8]. Central to this response is an imbalance between anti-angiogenic and pro-angiogenic factors. Soluble fms-like tyrosine kinase-1 (sFlt-1), an anti-angiogenic protein that sequesters circulating vascular endothelial growth factor (VEGF) and PlGF, is over-expressed by the ischaemic placenta, while free PlGF falls. The resulting reduction in VEGF/PlGF bioavailability disrupts the maintenance of a healthy endothelium throughout the maternal vasculature [47,48].

Maynard et al. [47] showed that excess sFlt-1 alone can reproduce the hypertension, proteinuria and glomerular endotheliosis of preeclampsia in animal models, and Levine et al. [48] showed that circulating angiogenic factors are perturbed weeks before clinical onset. These observations underpin the clinical use of the sFlt-1/PlGF ratio, which was validated in the PROGNOSIS study by Zeisler et al. [49] and on an automated immunoassay platform by Verlohren et al. [50]. The ratio is used both to predict short-term progression and, given its high negative predictive value, to rule out disease in women with suspected preeclampsia [49,50]. Endothelial dysfunction, oxidative stress and impaired nitric-oxide-mediated vasodilatation are the unifying mechanisms that connect the maternal syndrome across organ systems [24].

The clinical phenotype of preeclampsia is, in effect, read out through several microvascular beds simultaneously. Glomerular endotheliosis manifests as proteinuria. Cerebral endothelial dysfunction and impaired autoregulation underlie the visual aura and headache and, at the extreme, posterior reversible encephalopathy syndrome (PRES) and eclamptic seizures [24]. The choroidal and retinal circulations are subject to the same vasoconstrictive and permeability-altering forces. The retinal arterioles, in particular, share embryological origin, calibre and autoregulatory behaviour with the cerebral microvasculature, which is otherwise inaccessible via direct observation. This is the basis for the long-standing interest in the ophthalmic and central retinal circulation as a proxy for intracranial vascular status [27,28]. Importantly, the choroid is a high-flow bed under autonomic and humoral control, and choroidal thickness and flow may be especially sensitive to the angiogenic imbalance of preeclampsia, providing a mechanistic rationale for the OCT and OCTA findings discussed below.

Two features of this framework are pivotal for interpreting the retinal literature. First, if retinal microvascular change is driven by the same circulating anti-angiogenic milieu that precedes clinical preeclampsia, retinal biomarkers might, in principle, be detectable before the diagnosis is made. This is the property that would make them useful for prediction rather than mere confirmation [27,28]. Second, because hypertension itself, independent of preeclampsia, narrows retinal arterioles, disentangling the contribution of the preeclamptic process from that of blood pressure per se is a recurrent and unresolved methodological challenge [28]. Studies that adjust vessel calibre for mean arterial pressure, or that compare preeclampsia against gestational and chronic hypertension rather than against normotensive controls alone, are, therefore, especially informative [27,28].

The retinal–cerebral analogy deserves particular emphasis because it explains why ocular vascular markers attract interest beyond the eye. The retinal and cerebral microcirculations share anatomical calibre, embryological origin, the presence of a blood–tissue barrier, and broadly similar autoregulatory behaviour, such that the retina is widely regarded as an accessible surrogate for the otherwise hidden intracranial vasculature [25,27,51]. In preeclampsia, failure of cerebral autoregulation in the face of acute hypertension is thought to underlie hyperperfusion, vasogenic oedema and the clinical picture of PRES [24]. The visual symptoms that frequently herald severe disease reflect involvement of the occipital cortex and the posterior visual pathways as well as the retina itself [24]. Markers that probe the ophthalmic and central retinal circulation, whether by Doppler interrogation of flow or by photographic measurement of calibre, therefore, carry a plausible claim to index cerebrovascular as well as retinal risk. This is one reason the ophthalmic artery has attracted sustained investigation as a screening adjunct [32,52].

Beyond the angiogenic axis, preeclampsia is characterised by systemic oxidative stress, an exaggerated maternal inflammatory response, and activation of the complement and coagulation cascades, all of which converge on endothelial injury and increased vascular permeability [7,8,24]. These processes are not specific to any single organ, which is precisely why a peripheral, directly observable vascular bed might capture the systemic state. At the same time, this non-specificity is a double-edged sword for biomarker development. Retinal changes that reflect generalised endothelial dysfunction may not distinguish preeclampsia from other states of maternal cardiovascular strain. Moreover, constitutional or pre-existing microvascular characteristics, themselves risk factors for preeclampsia, may confound cross-sectional comparisons. Recognising preeclampsia as a heterogeneous syndrome with distinct early- and late-onset phenotypes, each with differing degrees of placental and maternal cardiovascular contribution, is, therefore, important when interpreting and designing retinal studies [11,53].

The choroid deserves separate mechanistic consideration, because it is the ocular structure most often implicated in preeclampsia, both in depth-resolved imaging studies and in the exudative retinal detachments of severe disease. The choroid is among the most highly perfused tissues in the body, supplied by fenestrated capillaries and richly innervated by autonomic fibres. Its blood flow is governed by humoral vasoactive factors and sympathetic tone rather than by the tight autoregulation that characterises the retinal circulation proper [30]. These features make the choroid especially susceptible to the circulating anti-angiogenic and vasoconstrictive milieu of preeclampsia. Reduced bioavailability of VEGF and PlGF, both of which are trophic for fenestrated endothelium, is compounded by heightened sensitivity to vasoconstrictors. Together, these changes provide a plausible explanation for the reduced choriocapillaris flow and attenuated pregnancy-related choroidal thickening reported in some preeclamptic cohorts, and for the choroidal ischaemia that underlies exudative retinal detachment in severe disease [36,47,54,55,56]. Other cohorts, however, report choroidal thickening (Section 6). The choroid may, therefore, be the retinal compartment in which the systemic angiogenic imbalance is most faithfully and earliest expressed, a hypothesis that depth-resolved imaging is now well placed to test.

The mechanism-level correspondence between the systemic angiogenic and endothelial disturbances of preeclampsia and the specific retinal compartments they affect, together with the heterogeneous empirical findings reported across imaging modalities, is synthesised in Figure S3.

## 3. Retinal Features, Physiological Adaptation and Confounding

### 3.1. Retinal Features Assessed in Preeclampsia

**Figure 2:**
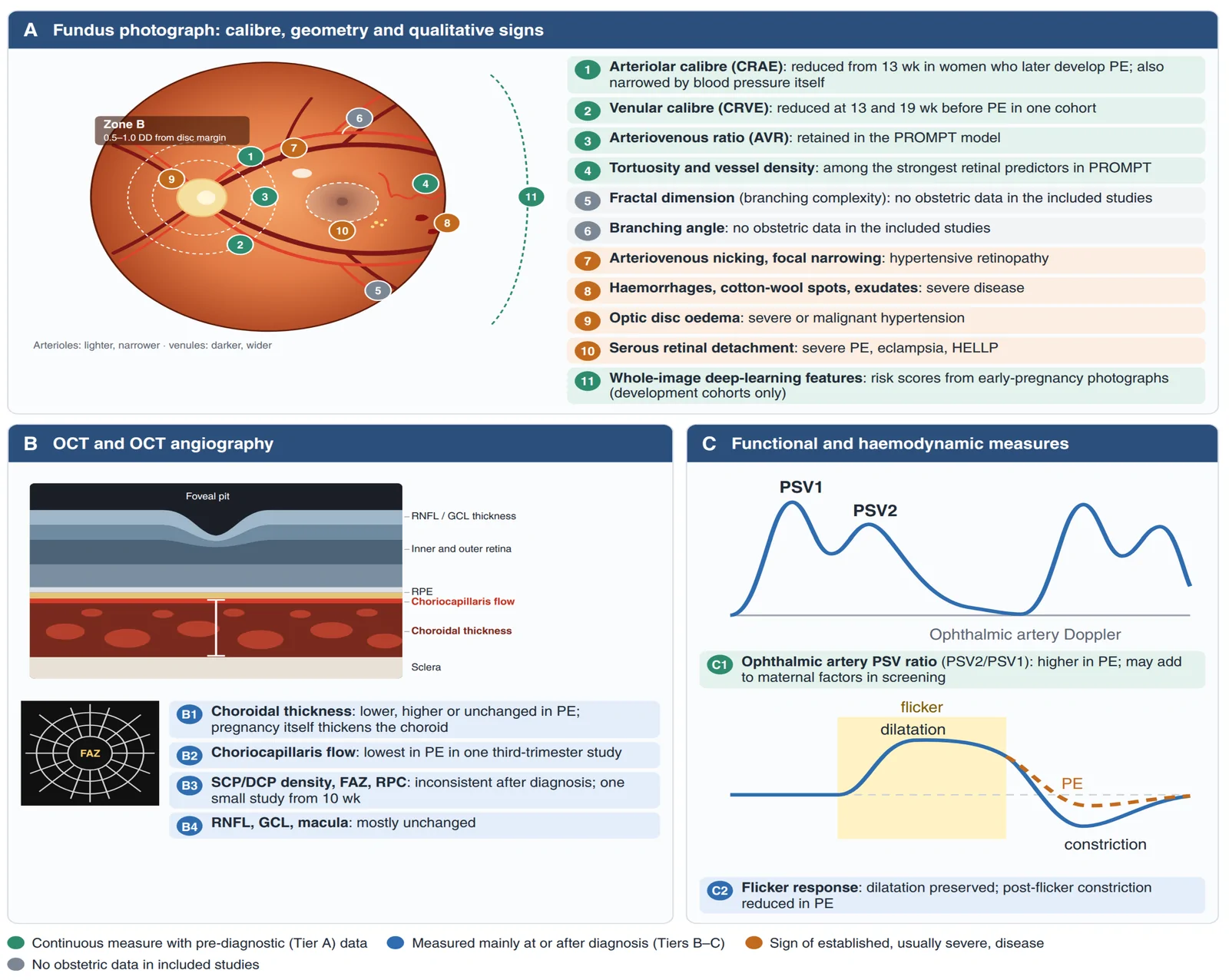
Retinal and ocular vascular features assessed in preeclampsia and their proposed relevance. (**A**) Fundus photography: calibre is measured in the 0.5–1.0-disc-diameter zone from the optic disc margin and summarised as the central retinal arteriolar and venular equivalents and the arteriovenous ratio. Geometric descriptors include fractal dimension, tortuosity and branching angle, whereas the qualitative signs of hypertensive retinopathy and serous retinal detachment mark established, usually severe, disease. (**B**) Optical coherence tomography (OCT) and OCT angiography (OCTA): retinal layer thickness, superficial and deep capillary plexus vessel density, foveal avascular zone, radial peripapillary capillary density, choriocapillaris flow and choroidal thickness. (**C**) Functional and haemodynamic measures: flicker-induced vasomotor response and ophthalmic artery Doppler peak systolic velocity ratio. Colour coding indicates the predominant evidence tier and the consistency of findings in preeclampsia (Section 4, Section 5, Section 6, Section 7 and Section 8). The schematic is illustrative and not to scale. AVR, arteriovenous ratio; CRAE, central retinal arteriolar equivalent; CRVE, central retinal venular equivalent; DCP, deep capillary plexus; FAZ, foveal avascular zone; GCL, ganglion cell layer; PSV, peak systolic velocity; RNFL, retinal nerve fibre layer; RPC, radial peripapillary capillaries; SCP, superficial capillary plexus.

The retinal and ocular features studied in preeclampsia differ in the vascular compartment they interrogate, the technology they require and the stage of disease at which they become abnormal (Figure 2). Fundus photography captures the calibre and geometry of the larger retinal arterioles and venules, summarised as the central retinal arteriolar and venular equivalents, the arteriovenous ratio, fractal dimension, tortuosity and branching angle. It also records the qualitative signs of hypertensive retinopathy: focal and generalised arteriolar narrowing, arteriovenous nicking, haemorrhages, cotton-wool spots, hard exudates and optic disc oedema [57,58,59]. OCT and OCTA resolve the retinal layers, the superficial and deep capillary plexuses, the foveal avascular zone (FAZ), the radial peripapillary capillaries, the choriocapillaris and the choroid, from which thickness, vessel density, flow and avascular-zone metrics are derived [30,60]. Dynamic vessel analysis quantifies flicker-induced vasomotor responses as a functional index of retinal endothelial function [61], and Doppler ultrasound of the ophthalmic and central retinal arteries measures upstream flow velocities and resistance [32,52]. Deep learning models, finally, may draw on the entire fundus image, including features not captured by any predefined metric [62]. The qualitative signs are, in general, markers of established and often severe disease, whereas the continuous quantitative and learned features are the only candidates for detecting the subclinical, pre-diagnostic change on which prediction depends.

### 3.2. Physiological Change in Pregnancy and Confounding Factors

Interpreting any retinal measurement in preeclampsia requires knowledge of how the ocular circulation behaves in healthy pregnancy. Lupton et al. [63] showed that retinal arteriolar and venular calibre widen between 13 and 19 weeks of gestation and return towards baseline by term, mirroring the mid-trimester fall and later recovery of mean arterial pressure. Kiziltunc et al. [60] found OCTA vessel density in the parafoveal and perifoveal superficial plexus and in the peripapillary capillaries to be higher in pregnant than in non-pregnant women. In a meta-analysis of 26 studies, Larsen et al. [64] found subfoveal choroidal thickness to be on average 31 µm greater in pregnancy, with a decrease of approximately 42 µm from the third trimester to the postpartum period. In a trimester-resolved OCTA study, Pehlivan et al. [38] similarly described a biphasic course of retinal capillary density across healthy gestation. Pregnancy itself, therefore, alters the very parameters under study. Comparisons with non-pregnant women, or between groups imaged at different gestational ages or postpartum intervals, can, thus, generate differences unrelated to preeclampsia.

Several maternal and technical factors also influence retinal measurements and are unevenly distributed between women with and without preeclampsia. Blood pressure is the dominant determinant of retinal arteriolar calibre [27,28,65]. An apparent effect of preeclampsia may, therefore, simply reflect higher pressure, unless calibre is corrected for mean arterial pressure or women with preeclampsia are compared with women with gestational or chronic hypertension. Age, body mass index and smoking are associated with retinal vessel calibre in the general population [28]. In the principal longitudinal cohort, that of Lupton et al. [66], the women who developed preeclampsia were older and had a higher body mass index than those who did not. Diabetes alters the retinal microvasculature [67]. In pregnancy, Phillipos et al. [37] found gestational diabetes to be independently associated with arteriolar narrowing of about 12 µm after adjustment for age, mean arterial pressure, hypertension and smoking, so coexisting diabetes is a potential confounder. Refractive error and axial length influence measured vessel calibre through ocular magnification [28] and are routinely adjusted for in analyses of choroidal thickness, as in the study of Garg et al. [68]. The diurnal variation in choroidal thickness has also led some investigators, such as Lee et al. [69], to standardise the time of acquisition. Finally, absolute values depend on the camera or OCT device, scan size, segmentation algorithm and analysis software, which limits comparability across studies [30,31,59]. Few obstetric studies have addressed more than two or three of these factors, and none has addressed them all. The confounders accounted for in each key study are, therefore, reported alongside its results (Table 1 and Table 2).

## 4. Fundoscopy and Hypertensive Retinopathy: The Qualitative Era

Direct ophthalmoscopy in pregnancy hypertension has a long clinical pedigree. The ocular manifestations described in preeclampsia and eclampsia range from focal and generalised arteriolar narrowing to retinal haemorrhages, cotton-wool spots, optic disc oedema and, in severe disease, exudative (serous) retinal detachment [24,30]. In an early prospective, controlled and masked study of 56 women, Jaffe and Schatz [70] found that a reduced arteriole-to-vein ratio and focal arteriolar constrictions were associated with severe preeclampsia. None of the women, however, had haemorrhages, cotton-wool spots, exudates or signs of choroidal ischaemia. In that study, retinal arteriolar changes tracked the systemic severity of disease. The clinical classification of the hypertensive disorders of pregnancy proposed by Davey and MacGillivray [71], however, relied solely on blood pressure and proteinuria. Conceptually, the fundus was treated as a severity gauge: the more florid the retinopathy, the more advanced the maternal vascular compromise.

The most influential hypertensive retinopathy grading systems were the Keith–Wagener–Barker and modified Scheie classifications and, later, the simplified mild/moderate/malignant schema of Wong and Mitchell [57]. These systems were developed largely in non-pregnant populations but were readily applied to obstetric patients. The pathophysiological sequence they describe (vasoconstrictive arteriolar narrowing, followed by a sclerotic and then an exudative phase with breakdown of the blood–retinal barrier) maps closely onto the accelerated, pressure-driven microvascular injury seen in severe preeclampsia. Crucially, large population studies subsequently linked retinal signs to systemic outcomes such as stroke, incident hypertension and coronary heart disease, validating the principle that the retinal phenotype carries prognostic information beyond the eye [27,28,57,72,73].

The limitations of qualitative fundoscopy as a screening tool, however, are well recognised. Grading is subjective and shows substantial inter-observer variability; many of the classic signs appear only in established, often severe, disease and, therefore, offer little for early prediction, and the technique depends on a trained examiner and clear media. In normotensive pregnancy and in mild preeclampsia, the fundus is frequently unremarkable. In terms of Figure S2, the fundoscopic literature on preeclampsia consists almost entirely of descriptive Tier B and Tier C evidence. These constraints motivated the shift from subjective grading to the objective, reproducible quantification of the retinal vasculature described in the next section.

## 5. Quantitative Retinal Vessel Analysis

### 5.1. Methodology

Quantitative retinal vessel analysis derives reproducible geometric measurements from standardised retinal fundus photographs, most commonly centred on the optic disc. Vessels crossing a defined zone around the disc are measured and combined, using the revised Knudtson–Parr–Hubbard formulae, into the central retinal arteriolar equivalent (CRAE) and central retinal venular equivalent (CRVE); their ratio yields the arteriovenous ratio (AVR) [58,74]. Generalised arteriolar narrowing is reflected by a reduced CRAE or AVR. Semi-automated platforms such as the Singapore I Vessel Assessment (SIVA) and the IVAN software standardise these measurements and extend them to additional geometric descriptors, namely, fractal dimension (a summary of branching complexity), tortuosity, branching angle and vessel wall-to-lumen indices [59]. Normative ranges and the systemic, environmental and genetic determinants of retinal calibre have been characterised in large epidemiological cohorts, providing the reference framework against which pregnancy studies are interpreted [28,65,67,75].

In the obstetric context, an essential refinement is the correction of vessel calibre for mean arterial pressure, since blood pressure is itself a powerful determinant of arteriolar diameter. Failure to account for this confounder risks attributing to preeclampsia changes that simply track rising blood pressure. The most informative studies, such as those by Lupton et al. [63,66], therefore, report pressure-adjusted calibres and, ideally, follow women longitudinally so that the temporal relationship between retinal change and clinical diagnosis can be established. The other maternal and technical determinants of retinal calibre are considered in Section 3.2.

Beyond calibre, the geometric descriptors quantified by modern software warrant a brief explanation because they capture complementary aspects of microvascular health. Fractal dimension summarises the space-filling complexity of the retinal vascular tree, and a lower fractal dimension denotes a sparser, less branched network. Tortuosity quantifies the deviation of vessels from a straight course and is sensitive to haemodynamic and structural change. The branching angle and the ratio of vessel wall thickness to lumen diameter provide further indices of arteriolar remodelling. In a population-based study of non-pregnant adults, Cheung et al. [65] found fractal dimension, tortuosity and branching asymmetry to be independently associated with blood pressure. These metrics are appealing because they are continuous, observer-independent once the software is calibrated, and potentially more sensitive than calibre alone. However, they are also susceptible to image quality artefacts, differences in field of view and grading-zone definition, and inter-software variability. These factors constrain comparability between studies and underscore the need for standardised acquisition and reporting [58,59,74].

### 5.2. Retinal Calibre and Vascular Function Before and After Diagnosis

Pre-diagnostic (Tier A) evidence for retinal calibre rests principally on a single longitudinal cohort. In that Australian study, Lupton et al. [66] imaged 92 women at 13, 19, 29 and 38 weeks of gestation. In those who developed preeclampsia, the central retinal arteriolar equivalent corrected for mean arterial pressure was reduced at 13, 19 and 38 weeks, and the corresponding venular equivalent at 13 and 19 weeks. The analyses were adjusted for age and body mass index, both of which were higher in the preeclampsia group. These differences preceded the clinical onset of disease by several months and suggest early microvascular constriction. However, the cohort was small and came from a single centre. Moreover, differences in mean calibre between groups do not in themselves establish predictive accuracy, so the study demonstrates temporality rather than predictive performance. In a separate study, Lupton et al. [63] also characterised the physiological trajectory of retinal arteriolar and venular calibre across healthy pregnancy, establishing the normal pattern against which preeclamptic deviation can be judged.

Most other calibre and vascular function studies were performed at or after diagnosis (Tier B) or long after delivery (Tier D). In the cross-sectional study of Phillipos et al. [37], women with pregnancy-associated hypertension had markedly narrower arterioles (mean: 139.9 vs. 159.9 µm) and venules (211.7 vs. 232.8 µm) than normotensive women in the third trimester. These comparisons, however, were unadjusted and did not distinguish preeclampsia from gestational hypertension. Using dynamic vessel analysis, Bruckmann et al. [61] found that flicker-induced dilatation was preserved in women with preeclampsia, whereas the arteriolar constriction that normally follows the flicker stimulus was reduced both during pregnancy and postpartum. The analyses were adjusted for age, body mass index, mean arterial pressure, baseline diameter and family history, and the authors attributed the pattern to microvascular endothelial dysfunction. In the population-based Generation R study, Benschop et al. [76] performed retinal imaging approximately six years after the index pregnancy. Women with previous preeclampsia had narrower arterioles (−0.40 standard deviation scores; 95% confidence interval [CI]: −0.62 to −0.19), as did those with previous gestational hypertension (−0.20; −0.34 to −0.05). These associations were independent of age, ethnicity, education, smoking and pre-pregnancy body mass index and situate the obstetric findings within a life-course cardiovascular framework. Because pre-pregnancy retinal data were unavailable, however, these differences cannot be attributed to the pregnancy rather than pre-existing vascular characteristics.

Subsequent work has extended the window of interest to the preconception period. In a prospective cohort of 369 women, Li et al. [77] found that generalised maternal retinal arteriolar narrowing measured before pregnancy was associated with lower foetal abdominal circumference z-scores across gestation (−0.34; 95% CI: −0.66 to −0.03). This finding reinforces the notion that a constitutionally compromised maternal microvasculature predisposes to placentally mediated complications. Because the study examined foetal growth rather than preeclampsia, however, it provides indirect rather than direct predictive evidence. Collectively, these data support a real but modest signal: retinal calibre differs, on average, between groups, and some differences emerge early. The magnitude of the difference at the individual level is small, however, and overlapping distributions limit discrimination when calibre is used as a stand-alone predictor.

A related and clinically mature line of investigation concerns the ophthalmic artery, interrogated by Doppler ultrasound rather than by photography. The ophthalmic artery is a readily accessible proxy for the cerebral circulation, and Lau et al. [52] showed that its peak systolic velocity ratio distinguishes preeclampsia from chronic and gestational hypertension. In their prospective cohort of 160 women attending a specialist hypertension clinic (50 with preeclampsia, 54 with gestational hypertension and 56 with chronic hypertension), preeclampsia was associated with a higher ratio. This association was independent of maternal characteristics, mean arterial pressure and antihypertensive treatment. Because the measurements were obtained in women already presenting with hypertension, this is Tier B evidence. In a systematic review and meta-analysis conducted within our own department, Sapantzoglou et al. [32] pooled six observational studies (10,408 women) and found that adding ophthalmic artery Doppler to maternal factors improved discrimination for preeclampsia. The pooled area under the curve increased from 0.71 (95% CI: 0.68–0.75) to 0.79 (0.72–0.87), with a value of 0.88 (0.82–0.94) for preterm preeclampsia. Overlapping confidence intervals, however, temper the strength of this conclusion and argue for further focused study. Ophthalmic artery Doppler and photographic QRVA are complementary: the former captures haemodynamic resistance in a vessel that mirrors intracranial flow, while the latter captures structural calibre and geometry in the terminal retinal bed.

Taken together, the QRVA literature supports several measured conclusions. There is a reproducible, biologically coherent association between retinal arteriolar and venular calibre and the hypertensive disorders of pregnancy [37,66,76]. In the cohort of Lupton et al. [66], at least some of this signal appeared before clinical diagnosis when calibre was corrected for blood pressure and measured longitudinally. The associations are, nonetheless, modest in magnitude and substantially overlapping at the individual level, so calibre alone is unlikely to function as a stand-alone screening test. The value of QRVA is more plausibly realised either as one input among several in a multivariable model or, increasingly, as a set of features to be learned and weighted automatically by the deep learning approaches discussed below. The heterogeneity of devices, grading zones and analysis software across the obstetric QRVA studies remains the principal barrier to pooling results and to defining clinically actionable thresholds, a limitation it shares with the OCT and OCTA literature [31]. The design, timing, adjustment for confounders and limitations of the key studies are summarised in Table 1.

**Table 1:** Quantitative retinal vessel, retinal vascular function and ophthalmic artery Doppler studies in pregnancy and preeclampsia, by evidence tier.

Study	Tier	Design; Setting; Participants (n)	Timing of Assessment	Modality and Metric	Principal Quantitative Findings	Confounders Addressed	Key Limitations; Overall Concern (Tool)
Lupton et al. (2013) [63]	Ref.	Prospective cohort; healthy pregnant women (53)	13, 19, 29 and 38 wks; 6 months postpartum	Fundus photography; CRAE and CRVE (uncorrected and MAP-corrected)	Arteriolar and venular calibre widened between 13 and 19 wks and returned towards baseline by term, mirroring the fall and recovery of MAP	MAP correction; parity	Small; healthy women only; no PE outcome. Moderate (NOS)
Lupton et al. (2013) [66]	A	Prospective cohort; single tertiary hospital, Sydney; 92 women (PE vs. normotensive)	13, 19, 29 and 38 wks (before diagnosis)	Fundus photography; MAP-corrected CRAE and CRVE	In women who developed PE: CRAE reduced at 13 (*p* = 0.03), 19 (*p* = 0.007) and 38 wks (*p* = 0.03); CRVE reduced at 13 (*p* = 0.04) and 19 wks (*p* = 0.001)	MAP correction; age; BMI	Small single-centre cohort; group differences only (predictive accuracy not established); repeated comparisons. Moderate (NOS)
Li et al. (2024) [77]	A	Prospective preconception cohort; 369 women	Preconception	Fundus photography; generalised retinal arteriolar narrowing	Narrowing associated with lower foetal abdominal circumference z-score across pregnancy (−0.34; 95% CI: −0.66 to −0.03) and slower AC and FL growth from 24–28 to 32–34 wks	Fully adjusted model (covariates as specified in the original report)	Foetal growth, not PE, was the outcome (indirect evidence); single hospital. Moderate (NOS)
Phillipos et al. (2023) [37]	B	Cross-sectional; single centre, Melbourne; PAH: 35, GDM: 68, SGA: 31, and controls: 27	Third trimester (PAH after diagnosis)	Fundus photography; CRAE and CRVE; flicker response (subset)	PAH vs. controls: arterioles: 139.9 vs. 159.9 µm and venules: 211.7 vs. 232.8 µm (both *p* < 0.0001); GDM independently associated with narrower arterioles (−12.3 µm; 95% CI: −17.8 to −6.9)	PAH comparison unadjusted; GDM model adjusted for age, MAP, hypertension and smoking	PE not distinguished from gestational hypertension; single BP reading; small control group; high (NOS)
Bruckmann et al. (2015) [61]	B, D	Prospective observational; two German centres; PE: 34, matched normal pregnancies: 45, and non-pregnant: 22	Pregnancy (after diagnosis) and postpartum	Dynamic vessel analysis; flicker-induced dilatation and post-flicker constriction	Dilatation preserved; arteriolar constriction and constriction amplitude reduced in PE during pregnancy and postpartum	Age, BMI, MAP, baseline diameter, and family history; matching	Small; established disease only; moderate (NOS)
Benschop et al. (2017), Generation R [76]	D	Population-based prospective cohort; Rotterdam; 3391 women (PE: 63; GH: 145)	6.2 years (90% range: 5.7–7.4) after the index pregnancy	Fundus photography (left eye); CRAE and CRVE (SD scores)	Previous PE: arterioles: −0.40 SDS (95% CI: −0.62 to −0.19); previous GH: arterioles: −0.20 SDS (−0.34 to −0.05) and venules: +0.22 SDS (0.07 to 0.36)	Age, ethnicity, education, smoking, pre-pregnancy BMI, and fellow-vessel calibre; MAP examined as mediator	No pre-pregnancy retinal data; imaging missing in 33.6%; small PE group; moderate (NOS)
Lau et al. (2022) [52]	B	Prospective cohort; specialist hypertension clinic; PE: 50, GH: 54, and CH: 56	32^+0^–36^+6^ wks, at assessment for hypertension	Ophthalmic artery Doppler; PSV ratio	PE independently associated with a higher PSV ratio after adjustment (*p* = 0.0002)	Maternal characteristics, medical history, MAP, and antihypertensive treatment	Spectrum limited to women referred with hypertension; screening performance not assessed; moderate (QUADAS-2)
Sapantzoglou et al. (2025) [32]	A	Systematic review and meta-analysis of screening studies; 6 observational studies; 10,408 women	Antenatal screening	Ophthalmic artery Doppler added to maternal factors	Pooled AUC for PE: 0.71 (0.68–0.75) without vs. 0.79 (0.72–0.87) with ophthalmic artery Doppler; preterm PE: 0.88 (0.82–0.94); term PE: 0.76 (0.65–0.88)	Combined with maternal factors	Few studies; overlapping 95% CIs; moderate (AMSTAR 2)

AC, abdominal circumference; AMSTAR 2, A Measurement Tool to Assess Systematic Reviews 2; AUC, area under the receiver operating characteristic curve; BMI, body mass index; BP, blood pressure; CH, chronic hypertension; CI, confidence interval; CRAE, central retinal arteriolar equivalent; CRVE, central retinal venular equivalent; FL, femur length; GDM, gestational diabetes mellitus; GH, gestational hypertension; MAP, mean arterial pressure; NOS, Newcastle–Ottawa Scale; PAH, pregnancy-associated hypertension; PE, preeclampsia; PSV, peak systolic velocity; QUADAS-2, Quality Assessment of Diagnostic Accuracy Studies 2; SDS, standard deviation score; SGA, small for gestational age; wk, week. Tiers: A, imaging before diagnosis (prediction); B, at or after diagnosis (diagnostic discrimination); C, severity within established disease; D, postpartum or long-term follow-up; Ref., healthy pregnancy reference study. External validation is not applicable, because none of these studies developed a prediction model. Overall concern summarises the study-level appraisal detailed in Section 10.1.

## 6. Optical Coherence Tomography and OCT Angiography

Optical coherence tomography revolutionised retinal imaging by enabling micron-scale, cross-sectional, depth-resolved reconstruction of the retinal and choroidal layers without contrast. OCT angiography (OCTA) extends this capability to the microvasculature by detecting decorrelation in the OCT signal produced by moving erythrocytes. It generates non-invasive, three-dimensional maps of the superficial and deep retinal capillary plexuses and the choriocapillaris, together with quantitative metrics such as vessel density, the foveal avascular zone (FAZ) area, and choroidal thickness and flow indices [30]. Because OCTA avoids the intravenous fluorescein used in conventional angiography, it is well suited to repeated measurement in pregnancy, where the safety of contrast agents is uncertain [60].

With one exception, studies applying OCT and OCTA to preeclampsia have imaged women at or after diagnosis (Tier B), typically comparing women with established disease against normotensive pregnant and non-pregnant controls. The reported parameters include peripapillary and macular vessel density, FAZ area, retinal nerve fibre layer (RNFL) and ganglion cell layer thickness, subfoveal choroidal thickness, and choriocapillaris flow [60,68,78]. Choroidal measures have attracted most attention, but their direction is not consistent. Atas et al. [54] and Erkan Pota et al. [55] found choroidal thickness to be lower in preeclampsia than in healthy pregnancy, although in the former study, it remained greater than in non-pregnant women. In a prospective study of 15 women per group, Fayed et al. [36] found the parafoveal choriocapillaris flow index to be lowest in preeclampsia, intermediate in hypertensive women and highest in healthy third-trimester pregnancies. In contrast, Garg et al. [68] found that women with severe preeclampsia imaged in the first postpartum week had thicker choroids (425 vs. 354 µm) and a greater macular volume than normotensive postpartum controls. This comparison was adjusted for age, parity, body mass index, race and axial length. In a postpartum Doppler–OCTA study, Silverman et al. [79] found no difference in choriocapillaris vessel density. Reduced choroidal flow is mechanistically coherent with the angiogenic imbalance and vasoconstriction that characterise the disorder, whereas thickening may reflect oedema and increased vascular permeability. Differences in timing, severity, postpartum fluid shifts and devices are plausible explanations for the discrepancy.

Interpreting this body of work requires awareness of several technical caveats intrinsic to OCTA. Quantitative metrics depend heavily on the instrument, the scan dimensions (for example, 3 × 3 mm versus 6 × 6 mm fields), the segmentation algorithm and the vessel density definition employed, so that absolute values are not directly comparable across devices or studies [30,31]. Projection artefacts, in which superficial vessels are erroneously duplicated in deeper slabs, and motion artefacts can distort density and FAZ measurements [30]. The choroid poses difficulties: choroidal thickness exhibits marked diurnal variation, prompting some investigators to standardise the time of acquisition [69], and automated choroidal segmentation remains less robust than retinal segmentation. Pregnancy itself induces physiological haemodynamic and hormonal changes in the ocular circulation independent of preeclampsia, which is why the most rigorous studies include both healthy pregnant and non-pregnant comparison groups [60,64] (Section 3.2). These factors collectively inflate between-study heterogeneity and caution against over-interpreting any single small cohort.

The picture for inner-retinal and microvascular metrics is considerably less consistent. Some studies report reduced superficial and deep capillary plexus vessel density and an enlarged FAZ in preeclampsia, while others find no significant differences from controls [30,55,78]. Reports on the RNFL, ganglion cell layer, and macular and total retinal thickness are similarly divergent, with most finding broadly comparable values between groups [55,60,68,78]. Kitmiridou et al. [31] synthesised 24 studies of OCT, OCTA, fundus photography and central retinal artery Doppler without meta-analysis. They concluded that the choroidal thickness data were inconclusive, with thickness described as decreased, increased or unchanged across studies, and that the overall evidence for retinal structural and functional change in preeclampsia was uncertain. All included studies were judged to be at moderate (n = 8) or serious (n = 16) risk of bias using the Risk of Bias in Non-Randomised Studies of Interventions (ROBINS-I) tool. In a narrative review of seven OCTA studies, Christou et al. [30] reached a similar conclusion, attributing the inconsistency to differences in participant characteristics, disease definition and severity, devices, scan regions and sample size. A companion review of retinal imaging in gestational diabetes by Kitmiridou et al. [80] reached an analogously cautious verdict, underscoring that methodological heterogeneity, not modality, is the limiting factor.

An especially informative subset of studies has correlated chorioretinal measurements directly with the biochemical and clinical severity of preeclampsia. In a retrospective study of 52 women with preeclampsia, Lee et al. [69] found that an sFlt-1/PlGF ratio of 38 or more was associated with a thinner choroid in some subfields, whereas vessel density and FAZ did not differ. The ratio was inversely related to central subfield choroidal thickness in regression analysis, although not in simple correlation. This Tier C finding links the chorioretinal imaging phenotype to the angiogenic axis of the disease. However, the study had no normotensive controls and did not control for gestational age. Such correlations strengthen the biological plausibility of OCT-derived choroidal metrics as severity markers, even if they do not yet establish a predictive role. A recurring theme across the OCTA literature is that most acute changes resolve postpartum, although longitudinal data raise the possibility that some women retain long-standing microvascular alterations and a heightened risk of later ocular and cerebral small-vessel disease [31].

Pehlivan et al. [38] conducted the only included OCTA study to image women from the first trimester. They followed 22 women who developed preeclampsia, 23 with gestational diabetes and 24 with uncomplicated pregnancies at 10, 24 and 30 weeks of gestation. Healthy pregnancy showed an early rise and subsequent decline in capillary vessel and perfusion density. Preeclampsia and gestational diabetes, in contrast, showed exaggerated early hyperperfusion followed by a steeper and earlier decline, most evident in the deep capillary plexus and in the peripapillary and optic nerve head circulation. The FAZ enlarged with gestation in all groups, without between-group differences. These observations are the only available data suggesting that OCTA changes may be detectable before the clinical onset of preeclampsia. They come from small groups, however, and require replication, formal evaluation of predictive accuracy and adjustment for blood pressure and other confounders before any predictive inference can be drawn.

The question of reversibility is central to the predictive ambition of OCT and OCTA. If retinal and choroidal changes were purely a consequence of acute hypertension, they would be expected to normalise once blood pressure is controlled and the pregnancy is delivered. If, in contrast, they reflected a more durable maternal microvascular trait, they might be detectable before disease onset and persist afterwards. The available longitudinal data are mixed. Many studies document substantial postpartum recovery of choroidal thickness and vessel density metrics, consistent with a reactive process [31]. However, population evidence that women with a history of preeclampsia carry an elevated long-term risk of cardiovascular and cerebral small-vessel disease suggests that some microvascular signature may endure [27,81,82]. Reconciling these observations will require cohorts that image the same women before, during and after pregnancy with standardised protocols. Until then, the most defensible reading of the OCT/OCTA evidence is that the choroid is the most frequently affected structure, although the direction of change differs between studies. Inner-retinal microvascular metrics are inconsistently altered, and the predictive, as opposed to descriptive, value of these measurements remains unproven [31]. The key OCT and OCTA studies are summarised in Table 2.

**Table 2:** Optical coherence tomography (OCT) and OCT angiography (OCTA) studies in pregnancy and preeclampsia, by evidence tier.

Study	Tier	Design; Participants (n)	Timing of Imaging	Modality and Metrics	Principal Quantitative Findings	Confounders Addressed	Key Limitations; Overall Concern (Tool)
Kiziltunc et al. (2020) [60]	Ref.	Comparative; pregnant: 124 (248 eyes), non-pregnant: 40 (80 eyes)	During pregnancy	OCTA; macular and peripapillary vessel density	Parafoveal and perifoveal SCP, whole-disc and RPC vessel density higher in pregnancy; no correlation with gestational week	No PE group	Cross-sectional comparison; device-specific values; moderate (NOS)
Pehlivan et al. (2026) [38]	A, B	Prospective longitudinal; PE: 22, GDM: 23, and healthy pregnancy: 24; non-pregnant: 31	10, 24 and 30 wks	OCTA; SCP/DCP vessel and perfusion density, ONH/RPC, choriocapillaris, and FAZ	Healthy pregnancy: early rise and later decline in capillary density; PE and GDM: exaggerated early hyperperfusion, then steeper and earlier decline (DCP, ONH, and RPC); FAZ enlarged with gestation in all groups	Within-woman longitudinal design; GDM comparator group	Small groups; predictive accuracy not established; not yet replicated; moderate (NOS)
Atas et al. (2014) [54]	B	Comparative; PE: 27, healthy pregnancy: 25, and non-pregnant: 26	Pregnancy, after diagnosis	SD-OCT; macular, RNFL and choroidal thickness	Choroidal thickness differed across groups (*p* < 0.001): greatest in healthy pregnancy, lower in PE, and lowest in non-pregnant women; central macular thickness lower in both pregnant groups	None described	Small; no adjustment for BP, gestational age or axial length described; high (NOS)
Ciloglu et al. (2019) [78]	B	Comparative; PE: 55, healthy pregnancy: 43, and non-pregnant: 38	Pregnancy, after diagnosis	OCTA; foveal and parafoveal SCP/DCP density, RPC density, and RNFL	Deep parafoveal density lower in PE than in both control groups (*p* = 0.001); foveal density lower than in non-pregnant women; RPC density higher than in both control groups; peripapillary RNFL higher in both pregnant groups than in non-pregnant women	None described	Multiple sectoral comparisons without adjustment; high (NOS)
Erkan Pota et al. (2024) [55]	B	Single-centre comparative; PE: 27, healthy pregnancy: 30, and non-pregnant: 30	Pregnancy, after diagnosis	Swept-source OCTA; SCP/DCP/CC density, FAZ, RNFL, GCL, and choroidal thickness	Central foveal SCP and DCP density lower in PE than in both control groups (*p* = 0.03 and *p* < 0.01); choroidal thickness lower than in healthy pregnancy (*p* = 0.029); FAZ correlated with systolic BP; RNFL and GCL unchanged	Age-matched non-pregnant controls	Small; many parameters; no adjustment for BP described; high (NOS)
Fayed et al. (2023) [36]	B	Prospective; PE: 15, hypertensive: 15, and healthy: 15 (one eye each)	Third trimester, after diagnosis	OCTA; parafoveal choriocapillaris adjusted flow index	Flow index lowest in PE, intermediate in hypertensive and highest in healthy pregnancies	Hypertensive comparator group	Very small; hypertension subtype unspecified; moderate (NOS)
Garg et al. (2014) [68]	B	Incident case–control; severe PE: 15, normotensive postpartum: 15, and non-gravid: 19	Postpartum days 1–7 (median: 2)	EDI SD-OCT; subfoveal choroidal thickness and macular volume	Thicker choroid (425 vs. 354 µm; *p* = 0.021) and greater macular volume (9.0 vs. 8.7 mm^3^; *p* = 0.006) in severe PE	Age, parity, BMI, and race; axial length (choroid); inter-eye correlation	Small; severe PE only; early puerperal imaging; moderate (NOS)
Silverman et al. (2023) [79]	B	Consecutive cohort; PE: 22 (mild: 5; severe: 17), CH/GH: 7, and controls: 11	Within 72 h of delivery	Plane-wave Doppler (CRA, SPCA, and choroid) and OCTA vessel density	Lower flow resistance in severe PE; SPCA flow moderately correlated with CC and peripapillary density; OCTA density not different from controls	Hypertensive comparator group	Small; postpartum timing; moderate (NOS)
Lee et al. (2021) [69]	C	Retrospective; 52 women with PE (104 eyes) stratified by sFlt-1/PlGF ratio < 38 (20) or ≥38 (32)	After diagnosis (gestational age not controlled)	Swept-source OCT/OCTA; choroidal thickness, FAZ, vessel density, and GCL+	Choroid thinner with ratio ≥38 in some subfields; ratio inversely associated with central choroidal thickness in regression (β = −6.66; *p* = 0.01) but not in simple correlation; vessel density and FAZ unchanged	Afternoon imaging (diurnal variation); BP not associated with thickness	Retrospective; no normotensive controls; covariates unspecified; high (NOS)
Kitmiridou et al. (2026) [31]	B	Systematic review; 24 studies, mainly Tier B (search July 2025)	Various	OCT, OCTA, fundus photography, and central retinal artery Doppler	Choroidal thickness reported as decreased, increased or unchanged; OCTA metrics inconsistent; risk of bias moderate in 8 and serious in 16 studies (ROBINS-I)	Not applicable	No meta-analysis because of heterogeneity; low–moderate (AMSTAR 2)

BP, blood pressure; CC, choriocapillaris; CH, chronic hypertension; CRA, central retinal artery; DCP, deep capillary plexus; EDI SD-OCT, enhanced-depth-imaging spectral-domain OCT; FAZ, foveal avascular zone; GCL, ganglion cell layer; GDM, gestational diabetes mellitus; GH, gestational hypertension; NOS, Newcastle–Ottawa Scale; ONH, optic nerve head; PE, preeclampsia; PlGF, placental growth factor; RNFL, retinal nerve fibre layer; ROBINS-I, Risk of Bias in Non-Randomised Studies of Interventions; RPC, radial peripapillary capillaries; SCP, superficial capillary plexus; sFlt-1, soluble fms-like tyrosine kinase-1; SPCA, short posterior ciliary arteries; wk, week. Tiers are defined in Figure S2 and Table 1. Absolute values are device- and protocol-specific and are not comparable across studies. External validation is not applicable, because none of these studies developed a prediction model.

## 7. Severe Retinal Complications of Preeclampsia and Eclampsia

Although the predictive ambition of retinal imaging concerns subclinical change, the severe ocular complications of preeclampsia remain clinically important and illustrate the extremes of the same microvascular pathophysiology. Visual disturbances, including blurring, scotomata, photopsia and, occasionally, transient cortical blindness, are reported by a meaningful minority of women with severe preeclampsia and eclampsia [83,84]. They are recognised as warning features in clinical guidelines [4,5]. Most are transient and resolve postpartum, but they warrant prompt evaluation because they may herald cerebral involvement [83,84].

Exudative (serous) retinal detachment is the most striking retinal complication, arising from choroidal ischaemia and breakdown of the blood–retinal barrier with subretinal fluid accumulation [30,85]. It is typically bilateral and characteristically associated with severe preeclampsia, eclampsia and the HELLP syndrome [86]. Reassuringly, it usually resolves spontaneously with control of blood pressure and delivery, with good visual recovery in the majority of cases [85,86,87]. A number of case reports and small series document its presentation, imaging features on OCT, and benign course when managed conservatively [88,89,90]. Other recognised but rarer associations include Purtscher-like retinopathy, central retinal vein occlusion and retinal or vitreous haemorrhage, whereas cortical blindness reflects cerebral rather than retinal involvement [83,84].

From a predictive standpoint, these complications are of limited value, as they occur in established, often advanced, disease rather than antenatally (Tier C). They are, nonetheless, instructive, because the choroid is the principal site of injury in serous detachment. This observation converges with the OCT and OCTA evidence that choroidal thickness and flow are the most frequently altered retinal parameters in preeclampsia, even though their direction varies between studies. The severe end of the clinical spectrum, thus, corroborates, at the level of overt pathology, the subclinical choroidal signal that quantitative imaging attempts to detect.

From a clinical management perspective, the recognition of these complications matters for both reassurance and triage. New visual symptoms in a woman with preeclampsia should prompt urgent assessment of blood pressure and disease severity and consideration of expedited delivery [4,5]. The ophthalmic prognosis of the characteristic lesions, hypertensive retinopathy and exudative retinal detachment, is, nonetheless, generally favourable, with conservative management and control of the underlying disease sufficient in the great majority of cases [83,85]. OCT has become a useful bedside adjunct in this setting, allowing non-invasive confirmation of subretinal fluid and objective monitoring of its resolution without recourse to fluorescein angiography [85,87]. The same severe presentations also serve as a reminder that the boundary between the retinal and cerebral manifestations of preeclampsia is porous. Cortical visual disturbance arising from PRES can coexist with, or be mistaken for, retinal pathology, and the two may require different investigations [83]. This reinforces the value of structured ophthalmic and neurological evaluation when visual symptoms arise. These complications, and the type of evidence supporting them, are summarised in Table 3.

## 8. Artificial Intelligence-Assisted Retinal Imaging

### 8.1. From Handcrafted Metrics to Deep Learning

The application of deep learning to retinal fundus photographs has transformed ophthalmic image analysis over the past decade. In landmark studies by Gulshan et al. [91] and Ting et al. [92], convolutional neural networks trained on large, labelled datasets achieved expert-level performance in detecting diabetic retinopathy and other vision-threatening conditions, establishing the feasibility of automated retinal screening. A pivotal conceptual advance came when Poplin et al. [62] showed that deep learning models could infer systemic attributes directly from fundus photographs. These included age, sex, smoking status, blood pressure and cardiovascular risk, often predicted from features not previously appreciated by human graders. This “oculomics” paradigm, in which the retina serves as a biomarker for systemic disease, has since been extended to the estimation of systemic biomarkers by Rim et al. [93] and to cardiovascular risk assessment by Cheung et al. [94].

Two methodological strategies are evident in the literature. The first uses deep learning to automate and refine the extraction of interpretable vascular features (calibre, density, tortuosity, and fractal dimension) that are then entered into a conventional statistical or machine learning predictor. The second is end-to-end learning, in which the network maps the raw image directly to a risk estimate. This approach may capture predictive information that is invisible to handcrafted metrics, but at the cost of interpretability. Both approaches are now being applied to preeclampsia.

The broader oculomics field provides both encouragement and a cautionary template for the obstetric application. Deep learning systems have been developed to estimate systemic biomarkers, to quantify retinal vessel calibre automatically and relate it to cardiovascular risk, and to predict cardiovascular events directly from fundus photographs, and these capabilities have been reviewed extensively [93,94,95]. The trajectory of automated diabetic retinopathy screening is especially relevant. It progressed from proof-of-concept, through large multi-ethnic validation, to regulatory approval and real-world deployment, including in low-resource settings, precisely because the field converged on shared reference standards, large, labelled datasets and prospective validation [91,92,96,97]. The preeclampsia application is at a far earlier stage of this maturation curve. It also differs in an important respect. Diabetic retinopathy is itself a retinal disease with directly visible lesions, whereas preeclampsia is a systemic disorder whose retinal signature, if it exists, is subtle and partly subclinical. Detecting this signature is, therefore, closer in spirit to the oculomics task of inferring systemic state from a normal-appearing fundus than to lesion detection.

### 8.2. Deep Learning Prediction of Preeclampsia from Retinal Images

The first prospective evidence came from a cohort study by Zhou et al. [98] at the Shanghai First Maternity and Infant Hospital. Retinal fundus images were acquired with a non-mydriatic camera at the first antenatal visit, before 20 weeks of gestation. A deep learning hypertension detection model generated a continuous “fundus score” for each participant. Among 1138 women, 92 developed a hypertensive disorder of pregnancy (66 preeclampsia; 26 gestational hypertension). The fundus score was independently associated with preeclampsia (adjusted odds ratio: 2.58; 95% CI: 1.88–3.62), with a stronger effect in women with higher body mass indices. Combined with maternal risk factors, it achieved an area under the curve of 0.88 (95% CI: 0.83–0.93; sensitivity: 0.72 and specificity: 0.93) for predicting preeclampsia. By comparison, the area under the curve was 0.85 for the fundus score alone and 0.74 for risk factors alone [98]. These estimates, however, describe the performance of the model in the women from whom it was derived. Apart from sensitivity analyses of the association between the fundus score and preeclampsia, no internal validation procedure, calibration assessment or external validation was reported. In addition, the covariates of the adjusted analysis were not specified, and the image algorithm had been developed for hypertension detection in a general population rather than in pregnancy. These estimates, therefore, cannot be equated with the discrimination of the FMF first-trimester model, which was not evaluated in the same cohort.

More recent work has refined the vascular feature approach. In a single-centre study from China, Wu et al. [99] used a deep learning system to extract retinal vascular features from colour fundus photographs obtained at 11–13^+6^ weeks. These features were combined with maternal risk factors and mean arterial pressure in a logistic regression model, designated as PROMPT, for the prediction of preeclampsia and preterm preeclampsia. In a cohort of 1812 pregnancies, including 86 cases of preeclampsia (29 preterm), the model achieved a cross-validated area under the curve of 0.87 (95% CI: 0.83–0.90) for preeclampsia and 0.91 (0.85–0.97) for preterm preeclampsia. In comparison, the area under the curve for preeclampsia was 0.82 for a model based on maternal factors and mean arterial pressure and 0.74 for retinal features alone. The authors explicitly positioned the method as a non-invasive, accessible and cost-effective option for prenatal care, while acknowledging the need for larger and more diverse datasets and multicentre validation [99]. In an accompanying editorial, Heydari et al. [100] highlighted the potential of retinal screening as a low-cost, non-invasive strategy for the earlier detection of preeclampsia, while noting that larger and more robust studies are needed to validate these findings. The PROMPT model retained five retinal features: arteriovenous ratio, vessel skeleton density, tortuosity density, and the mean and variability of angle-based tortuosity. Tortuosity density, vessel skeleton density and arteriovenous ratio ranked among the five most influential predictors, together with mean arterial pressure and overweight. Tortuosity density and vessel skeleton density were higher, and angle-based tortuosity lower, in women who developed preeclampsia [99]. Such observations reinforce the shift away from single, handcrafted calibre metrics toward richer, automatically learned representations of retinal vascular geometry.

The architecture of the earlier model illustrates both the appeal and the opacity of the end-to-end approach. In the Shanghai cohort of Zhou et al. [98], the fundus score was generated by a convolutional network based on an Inception-ResNet design, originally trained to detect hypertension from fundus images. The network was then repurposed to yield a continuous probability that was treated as a predictor of subsequent preeclampsia. That a hypertension detection network transfers usefully to preeclampsia prediction is itself informative. It suggests that the model may be capturing blood-pressure-related microvascular features rather than a preeclampsia-specific signature. This observation connects the AI findings to the long-standing difficulty of separating the preeclamptic process from hypertension per se. It also raises the central interpretability question: if the predictive signal is largely a proxy for blood pressure and its vascular consequences, the incremental value over a directly measured mean arterial pressure must be demonstrated rather than assumed. Feature attribution methods such as saliency and class activation mapping, which highlight the image regions driving a prediction, are, therefore, not a cosmetic addition but a scientific necessity. They are needed both to build clinical trust and to test whether models are learning genuine vascular biology or exploiting confounders and dataset-specific artefacts.

These retinal models sit within a wider and rapidly expanding ecosystem of machine learning predictors for preeclampsia that draw on maternal characteristics, serum biomarkers, polygenic risk scores and cell-free RNA [33,101,102]. For example, the cell-free RNA signatures of Moufarrej et al. [103], which predict preeclampsia months before onset, have set a high bar for any new biomarker. In a systematic review and meta-analysis of 31 machine learning models from 26 studies, Liu et al. [33] reported a pooled area under the curve of 0.91 but a 95% prediction interval of 0.75–1.00. The review also found extreme heterogeneity, external validation for fewer than one-quarter of the studies and no pooled calibration data. The authors concluded that the high pooled discrimination largely reflects development rather than independent performance. This conclusion applies with equal force to the retinal-imaging models, one of which was included in that review.

### 8.3. Methodological Appraisal and Validation of AI Models

The enthusiasm warranted by these results must be tempered by methodological scrutiny. Table 4 sets out the validation conditions of the two retinal models against the items of TRIPOD + AI (Transparent Reporting of a Multivariable Prediction Model for Individual Prognosis or Diagnosis, updated for artificial intelligence) and PROBAST + AI [42,104]. Preeclampsia is a relatively low-prevalence outcome, so even models with high area-under-the-curve values may have modest positive predictive value in unselected populations, and class imbalance can inflate apparent performance if not handled appropriately. Neither study reported calibration, which determines whether predicted risks can be used to select women for prophylaxis [105], and neither model has been validated externally in a population distinct from its development cohort. Differences in camera hardware, field of view, image quality and labelling standards limit generalisability, and the “black-box” nature of end-to-end models complicates clinical trust [96]. Explainability techniques such as saliency mapping are, therefore, increasingly expected. Adherence to reporting and bias assessment frameworks, such as TRIPOD for prediction model reporting [106], PROBAST for risk-of-bias assessment [41] and ROBINS-I for non-randomised studies [107], remains inconsistent across the field. Such adherence should be a baseline expectation for future work.

**Figure 3:**
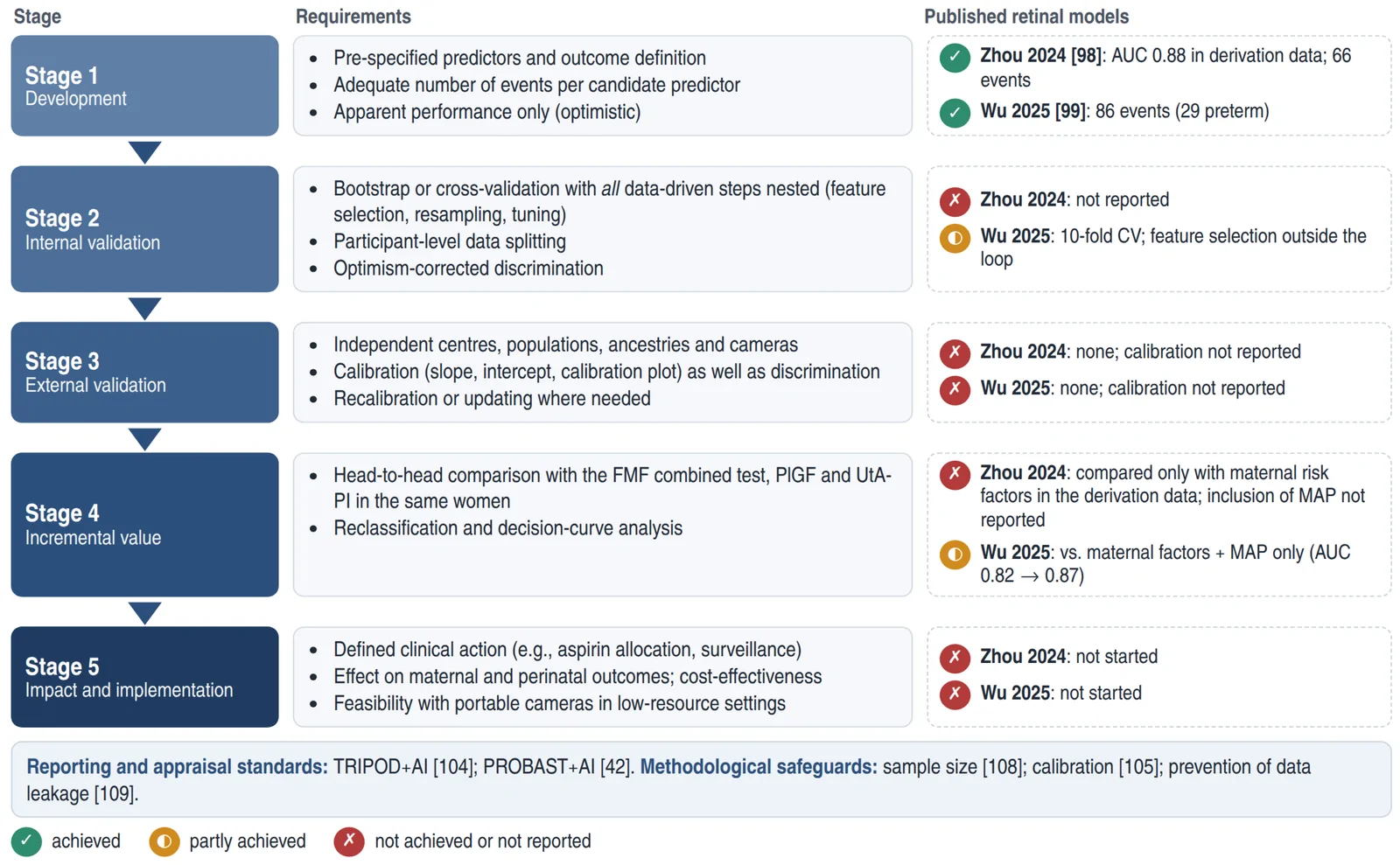
Stages of evidence required before a retinal prediction model for preeclampsia could be used in clinical practice, and the position of the published models. Stage 1, model development with apparent performance; stage 2, internal validation with all data-driven steps nested within resampling and participant-level data splitting; stage 3, external validation in independent populations, centres and cameras, including calibration; stage 4, head-to-head assessment of incremental value over established screening in the same women; and stage 5, impact and implementation studies. The published models of Zhou et al. [98] and Wu et al. [99] are at stage 1 or 2, and neither has reported calibration or external validation. FMF, Fetal Medicine Foundation; MAP, mean arterial pressure; PlGF, placental growth factor; PROBAST + AI, Prediction Model Risk of Bias Assessment Tool for Artificial Intelligence; TRIPOD + AI, Transparent Reporting of a Multivariable Prediction Model for Individual Prognosis or Diagnosis for Artificial Intelligence; UtA-PI, uterine artery pulsatility index.

Three further issues determine how far the reported discrimination can be trusted. First, the number of outcome events was small relative to the number of candidate predictors, with 66 cases of preeclampsia in the cohort of Zhou et al. [98] and 86 (29 preterm) in that of Wu et al. [99]. This increases the risk of overfitting and of imprecise performance estimates [108]. Second, the validation strategy matters. In the earlier study by Zhou et al. [98], the combined model appears to have been evaluated on the same data used to derive it, so that its area under the curve is an apparent estimate. In PROMPT, Wu et al. [99] used ten-fold cross-validation, but the retinal features were selected by least absolute shrinkage and selection operator (LASSO) and stepwise procedures before, rather than within, the cross-validation loop. Such pre-selection is a form of data leakage that typically inflates cross-validated performance [109]. Because only one image per woman was analysed in PROMPT, its cross-validation folds necessarily corresponded to participants [99]. This avoids the leakage that arises when images from the same woman or eye are split between training and test sets [109]. Future studies, which often acquire several images per participant, should report participant-level splitting explicitly. Third, incremental value was examined only partially. PROMPT improved discrimination over a model containing maternal factors and mean arterial pressure in the same cohort (0.87 vs. 0.82) [99]. In the earlier model, in contrast, the inclusion of blood pressure was not reported, leaving open the possibility that a network trained to detect hypertension largely captures blood-pressure-related information [98]. Neither model has been compared with the FMF algorithm, uterine artery Doppler or PlGF in the same women. Areas under the curve obtained in different cohorts, with different case-mix, outcome definitions, prevalence and gestational timing, cannot be compared directly. The stages of evidence required before clinical use, and the position of the current models along this pathway, are summarised in Figure 3.

## 9. Integration with Established Screening and Clinical Translation

If retinal imaging is to earn a place in antenatal care, it must demonstrate value relative to, or in combination with, the existing screening architecture. The established comparators, however, serve different purposes, and a retinal marker can be benchmarked only against a tool with the same intended use, population and timing (Table 5). The FMF first-trimester combined test is a population-screening tool applied at 11–13^+6^ weeks to select women for aspirin prophylaxis, and its detection rate for preterm preeclampsia is the relevant benchmark for first-trimester retinal models [12,15,16,17,18,19]. The sFlt-1/PlGF ratio, in contrast, is used from the late second trimester in women with suspected preeclampsia, chiefly to rule out disease within the following week [49,50]. In the same setting, Duhig et al. [110] showed that point-of-care PlGF-based testing shortened the time to clinical confirmation of preeclampsia. Neither the ratio nor PlGF-based testing is a first-trimester screening test, and fullPIERS, developed by von Dadelszen et al. [111], is a prognostic model for adverse maternal outcomes in established disease. The relevant question is rarely whether a retinal marker outperforms these in isolation, but whether it adds independent, incremental discrimination, ideally while reducing cost or extending reach. The ophthalmic artery Doppler meta-analysis of Sapantzoglou et al. [32] is instructive in this regard. The marker showed added value to established models, particularly for preterm preeclampsia, yet the overlapping confidence intervals illustrate how difficult it is to demonstrate robust incremental benefit over an already strong screening baseline. The retinal photography-based AI models of Zhou et al. [98] and Wu et al. [99] report area-under-the-curve values near 0.88 when combined with maternal factors, which is encouraging precisely because the photograph is cheap, rapid and operator-light. These values, however, were obtained in the development cohorts without external validation, and the models’ incremental value over the full FMF algorithm has not been established in head-to-head studies. The numerical similarity between these values and the discrimination of the FMF algorithm in its own validation studies should, therefore, not be read as equivalence.

The most compelling clinical argument for retinal imaging lies in scalability. The FMF algorithm requires trained sonographers and biochemical assays that are unavailable in much of the world [23]. Non-mydriatic fundus photography, in contrast, can be performed in minutes by non-specialist staff, increasingly with smartphone-based and portable retinal cameras. Bastawrous et al. [114] validated smartphone-based acuity testing in a community cohort in Kenya, Russo et al. [115] compared smartphone ophthalmoscopy with slit-lamp examination, and Bellemo et al. [116] validated deep learning diabetic retinopathy screening in a national programme in Zambia. Together, these studies demonstrate that the imaging-plus-AI pipeline can function outside tertiary centres. A preeclampsia risk estimate generated from a single photograph, interpreted automatically and transmitted by telemedicine, is an attractive prospect for community antenatal programmes, provided that the underlying models are externally validated in the relevant populations.

The health-economic logic reinforces this argument. The overwhelming majority of maternal deaths from preeclampsia occur in low- and middle-income countries [1,2]. In these settings, the screening infrastructure required by biomarker- and Doppler-based algorithms is least available, and the established benefit of early aspirin prophylaxis is, therefore, hardest to realise [23]. A low-cost, rapid, operator-light test that could direct aspirin to those most likely to benefit would address a genuine equity gap. Realising this, however, requires more than algorithmic accuracy. Whether retinal screening could be implemented at scale depends on several further factors. These include the marginal cost of camera hardware, the need for reliable image transmission and quality assurance, the governance of automated decision support, and the training and acceptability of the test among front-line antenatal staff. These implementation considerations have rarely been studied in the obstetric context and represent an important gap between proof-of-concept performance and deployable clinical tools.

Three practical conditions must, nonetheless, be met before such translation. First, acquisition and analysis protocols must be standardised, since current heterogeneity in cameras, fields and software is a major source of between-study inconsistency [30,31]. Second, models must be calibrated and externally validated across diverse ethnic and clinical populations, with transparent reporting per TRIPOD and TRIPOD + AI and bias assessment per PROBAST and PROBAST + AI [41,42,104,106]. Third, the intended clinical action must be defined, because a retinal risk score is useful only if it changes management, most plausibly by directing aspirin prophylaxis or intensified surveillance. Its deployment should ideally be evaluated against hard maternal and perinatal outcomes rather than discrimination metrics alone, in the manner of validated outcome prediction models such as fullPIERS [111].

It is useful to weigh the four retinal approaches against one another on the practical axes that determine clinical utility: accessibility, cost, reproducibility and the maturity of the supporting evidence. Qualitative fundoscopy is inexpensive and widely available but subjective, operator-dependent and informative mainly in established, severe disease, limiting its predictive role. Quantitative retinal vessel analysis offers objective, reproducible metrics from a standard photograph and a respectable evidence base, including longitudinal cohorts, but its effect sizes are modest, and its software and grading conventions are not standardised. OCT and OCTA provide the richest, depth-resolved structural and microvascular detail and have produced the most mechanistically coherent finding in the field, namely, choroidal involvement. However, they require costly hardware, are confined largely to small case–control studies, and yield inconsistent inner-retinal metrics. AI-assisted fundus photograph analysis combines the low cost and accessibility of photography with promising discrimination in two development cohorts, but it is the least mature in terms of external validation, calibration and transparency. No single modality currently dominates. The pragmatic implication is that photography-based QRVA and AI are the most plausible near-term screening tools by virtue of cost and scalability, whereas OCT/OCTA is presently better suited to mechanistic and severity research than to population screening. These comparisons are summarised in Table 6.

## 10. Evidence Gaps and a Future Research Agenda

Three fundamental uncertainties recur throughout the literature. The first is temporality. It remains unresolved whether measurable retinal changes precede the clinical onset of preeclampsia, and are, therefore, predictive, or whether they are a consequence of established hypertension and its cardiovascular sequelae. The longitudinal pressure-corrected calibre data of Lupton et al. [66] suggest that at least some arteriolar and venular change may antedate diagnosis. In the most recent systematic review of retinal imaging in preeclampsia, however, Kitmiridou et al. [31] judged the temporal relationship to be uncertain. The second is consistency: across OCT and OCTA parameters in particular, findings are frequently contradictory, with choroidal metrics described as decreased, increased or unchanged in different cohorts [31]. The third is quality: many studies are small case–control designs, judged to be at moderate or serious risk of bias, and few prediction models have been externally validated [31,33].

Taken together, the evidence reviewed here supports four principal conclusions. First, genuinely predictive (Tier A) evidence is limited. It is confined to a single small longitudinal calibre cohort [66], one small longitudinal OCTA study [38], a preconception cohort with a foetal growth outcome [77], an ophthalmic artery Doppler meta-analysis [32] and two single-centre deep learning development studies [98,99]. Second, the much larger body of OCT and OCTA work describes established disease and is internally inconsistent. Third, blood pressure, gestational physiology and other confounders are inadequately addressed in most studies. Fourth, the discrimination reported for retinal deep learning models has not yet been validated. These conclusions agree with the systematic review by Kitmiridou et al. [31], which found the evidence inconclusive and could not establish whether retinal changes precede or follow the onset of disease. These conclusions also agree with the narrative review of OCTA studies by Christou et al. [30] and with the meta-analysis of machine learning models by Liu et al. [33], which attributed high pooled discrimination largely to development-phase estimates. The present review extends these syntheses by locating each study on the timeline of pregnancy and by setting the validation status of the retinal models against the intended uses of established screening tools.

### 10.1. Study-Level Methodological Appraisal

The study-level appraisal of the key studies informing each tier, performed with the Newcastle–Ottawa Scale [39], QUADAS-2 [40], PROBAST [41] interpreted in the light of PROBAST + AI [42], and AMSTAR 2 [43], is summarised in Table 7. Three patterns emerge. First, concern was driven chiefly by confounding and selection in the observational imaging studies, most of which compared women with established disease against normotensive or non-pregnant controls without adjustment for blood pressure, gestational age or ocular factors. In the prediction model studies, concern was driven by the analysis domain, as neither study reported calibration or external validation. Second, the studies of least concern were those that addressed blood pressure directly, although all were small. Lupton et al. [66] corrected vessel calibre for mean arterial pressure, Lau et al. [52] and Bruckmann et al. [61] used statistical adjustment, and Fayed et al. [36] included a hypertensive comparator group. Third, among the systematic reviews, Kitmiridou et al. [31] judged the primary imaging literature to be at moderate or serious risk of bias. Liu et al. [33] found that the high pooled discrimination of machine learning models largely reflects development rather than independent performance. The predominant biases were spectrum bias from comparing established, often severe, disease with healthy controls, confounding by blood pressure and by the physiology of pregnancy, and optimism in model development. Each would tend to overstate rather than understate the predictive value of retinal markers.

### 10.2. Strengths of This Review

This review has several strengths. First, previous reviews have focused on clinical ocular manifestations [29], on a single modality [30,32] or on retinal structure without distinguishing the timing of assessment [31]. This review, in contrast, traces retinal assessment in preeclampsia across its entire methodological arc, from qualitative fundoscopy, through quantitative retinal vessel analysis and OCT/OCTA, to AI-assisted fundus photograph interpretation. It also classifies the evidence according to whether it bears on prediction, diagnosis, severity or long-term outcome. This breadth allows the relative maturity, reproducibility and translational readiness of each approach to be compared directly and places the obstetric evidence within the broader, more established fields of oculomics and automated retinal screening. Second, the literature search, eligibility criteria and evidence classification are reported in full in the Supplementary Materials. The key studies were also appraised individually with instruments matched to study design (Newcastle–Ottawa Scale, QUADAS-2, PROBAST and AMSTAR 2; Table 7), a degree of transparency that is uncommon among narrative reviews in this field. Third, this review is explicitly mechanism-anchored, mapping each imaging readout to the underlying placental–renal–retinal pathophysiology (Figure 1 and Figure S3). This helps distinguish findings that are biologically plausible and potentially causal from those that most likely reflect prevalent hypertension. Finally, by foregrounding methodological heterogeneity, risk of bias and the absence of head-to-head comparison with established biomarkers, this review provides a pragmatic roadmap for the studies that would be required to move retinal imaging toward clinical utility.

### 10.3. Limitations

Several limitations should, nonetheless, be acknowledged, and they operate at two levels: those of the present review and those of the underlying evidence base. As a narrative rather than systematic review, this work did not register a protocol or undertake a quantitative meta-analysis. Its study-level appraisal relied on domain-level judgements without numerical scoring and was confined to the key studies rather than every included source. This review is, therefore, susceptible to selection and interpretation bias in the choice and emphasis of the literature cited. The counts summarised in the flow diagram (Figure S1) should, accordingly, be read as a transparent account of the search rather than as a formal PRISMA yield. Language restriction to English-language publications may have introduced further selection bias and excluded relevant regional data. Three eligible studies were identified only during the revision process, indicating that the search, although structured, was not exhaustive. Finally, the appraisal relied on the information available in the published reports, and the definition and subtype of preeclampsia, the gestational age at imaging and the covariates adjusted for were inconsistently reported in the primary studies.

The limitations of the primary literature are more consequential. The obstetric studies are markedly heterogeneous in design, imaging device, gestational timing, diagnostic criteria for preeclampsia, comparison groups and analysis software, which precludes direct pooling and weakens any cross-study inference. Many of the most frequently cited findings derive from small, single-centre case–control studies that compare established disease with normotensive controls. Such designs are prone to spectrum bias and cannot establish whether retinal changes precede, and, therefore, predict, the clinical syndrome, rather than arising alongside prevalent hypertension. Quantitative vessel-calibre effects, although reproducible, are modest in magnitude and overlap substantially at the individual level, limiting stand-alone screening value. OCT and OCTA findings are additionally constrained by device- and software-dependent segmentation, inconsistent adjustment for blood pressure, refraction and axial length, and the physiological haemodynamic changes of normal pregnancy, all of which complicate interpretation. The artificial intelligence literature, while the most promising, is at particularly high risk of bias. Both published retinal models [98,99] are single-centre and were evaluated only in their development cohorts, without calibration or external validation, and their reporting adheres incompletely to prediction model standards (TRIPOD/PROBAST). Publication bias favouring positive results is likely across all modalities, and the rapidly evolving AI evidence, in particular, is liable to date quickly.

### 10.4. Future Directions and Clinical Translation

Translating retinal imaging into a usable preeclampsia-screening tool will require a coordinated research agenda. The single most important need is for adequately powered, prospective, multicentre cohorts that image women at defined gestational windows, ideally in the first or early second trimester before clinical disease, and follow them to delivery. Outcomes should be ascertained against contemporary ISSHP/ACOG criteria [4,5]. Such designs would allow genuinely predictive, rather than merely associative, signals to be identified. Acquisition protocols, devices and analysis software should be standardised, pre-specified and reported in full, ideally through multicentre consortia and shared, well-curated image repositories that permit external validation across devices, populations and ancestries. Studies should adjust for blood pressure and should report or account for gestational age at imaging, body mass index, diabetes, smoking, refraction or axial length, and device. Wherever possible, they should also compare preeclampsia against gestational and chronic hypertension rather than normotensive controls alone so that the disease-specific signal can be isolated from that of prevalent hypertension.

Prediction models should be developed and reported in full accordance with TRIPOD and TRIPOD + AI, and appraised with PROBAST and PROBAST + AI and, for non-randomised studies, ROBINS-I [41,42,104,106,107]. Derivation and validation data should be separated at the participant level, and all data-driven steps, including feature selection and resampling, should be nested within internal validation [109]. Sample sizes should be justified by formal criteria [108], and calibration should be reported as well as discrimination [105]. Models also require transparent handling of the maternal covariates on which much of the apparent performance depends, and explainability sufficient to establish that they are learning vascular biology rather than dataset-specific artefacts. Critically, retinal markers must be tested not in isolation but for incremental value over, and in combination with, the tools already in clinical hands. These are the sFlt-1/PlGF ratio, uterine artery Doppler and established first-trimester risk algorithms. Demonstrating that retinal data add information beyond these markers is the threshold for clinical relevance. Beyond discrimination, translational studies will need to address calibration to absolute risk, cost-effectiveness and the operational realities of portable fundus cameras and automated analysis in routine antenatal care. They should also evaluate prospectively whether retinal-informed screening ultimately improves maternal and perinatal outcomes. The field would also benefit from harmonised data sharing and reference standards analogous to those that accelerated automated diabetic retinopathy screening.

A particularly promising direction is multimodal integration. The predictive signals carried by retinal imaging, angiogenic biomarkers, uterine artery Doppler [112,113], maternal characteristics and emerging molecular markers such as cell-free RNA [101,103] are at least partly independent. Contemporary machine learning architectures are well suited to fusing heterogeneous data of this kind [33]. Retinal imaging need not be positioned as a competitor to the sFlt-1/PlGF ratio or the FMF model. A more realistic and clinically useful goal may be to determine the incremental information that a retinal photograph adds within an integrated, individualised risk model, and the point at which the added complexity ceases to justify the marginal gain. Such studies would also help resolve the temporality question, since serial multimodal assessment across gestation can establish whether the retinal signal leads or lags the biochemical and haemodynamic markers whose trajectories in preeclampsia are now well characterised. The convergence of inexpensive smartphone-based fundus photography with validated deep learning analysis is the development most likely to make retinal screening scalable. It has particular appeal for low-resource settings, where the burden of preeclampsia is greatest and access to biochemical testing and specialist ultrasound is most limited. Until prospective, externally validated, outcome-anchored evidence of this kind is available, retinal imaging is best regarded as a scientifically compelling and rapidly maturing research tool rather than a clinically validated screening test.

## 11. Conclusions

The retina offers a biologically rational and uniquely accessible window into the systemic microvascular dysfunction that defines preeclampsia. The trajectory from qualitative fundoscopy, through quantitative vessel analysis and OCT/OCTA, to AI-assisted image interpretation reflects a steady increase in objectivity, resolution and predictive ambition. The contemporary evidence is genuinely promising. One longitudinal cohort suggests that retinal arteriolar and venular narrowing may precede clinical disease. OCT and OCTA frequently implicate the choroid, although the direction of change is inconsistent, and the evidence derives almost entirely from established disease. Ophthalmic artery Doppler may add discriminatory information to maternal factors, and deep learning-based models applied to early-pregnancy fundus photographs report promising discrimination in their development cohorts. Yet the same evidence is heterogeneous, frequently underpowered, inconsistently adjusted for confounders and rarely externally validated, and it has not resolved whether retinal change is a cause, a correlate or a consequence of the maternal syndrome. Retinal imaging, particularly fundus photography coupled with artificial intelligence and portable cameras, has clear potential as a scalable adjunct to existing screening, with special appeal for low-resource settings. However, it is not yet ready for routine clinical use. Realising its potential will require standardised protocols, large prospective cohorts, rigorous and transparent external model validation, and a convincing demonstration of incremental value over the biomarkers already available to clinicians. Retinal vasculature is, for now, a compelling but unproven window for preeclampsia prediction, and its translation from association to clinical utility is the central task ahead.

## Figures and Tables

**Table 3:** Severe retinal and visual complications associated with preeclampsia, eclampsia and HELLP syndrome.

Complication	Typical Clinical Setting/Mechanism	Course and Management	Evidence Base (Tier)
Hypertensive retinopathy (arteriolar narrowing, haemorrhages, and cotton-wool spots) [57]	Pressure-driven microvascular injury; severity tracks systemic disease	Generally reversible with blood pressure control; a severity marker	Small prospective controlled study [70]; grading systems derived from non-pregnant populations [57] (C)
Exudative (serous) retinal detachment [85,87]	Choroidal ischaemia and blood–retinal barrier breakdown; severe PE/eclampsia/HELLP; usually bilateral	Usually spontaneous resolution after delivery and BP control; good visual prognosis	Retrospective series and case reports [85,86,87,88,89,90] (C)
Purtscher-like retinopathy [83]	Microvascular occlusion; reported in (pre)eclampsia	Variable visual recovery; supportive management	Review and isolated reports [83] (C)
Cortical visual disturbance/PRES correlates [83]	Cerebral endothelial dysfunction and failed autoregulation	Typically transient; warrants neuroimaging and urgent BP/seizure management	Reviews and case series [83,84] (C)

BP, blood pressure; HELLP, haemolysis, elevated liver enzymes, low platelets; PE, preeclampsia; PRES, posterior reversible encephalopathy syndrome. (C), evidence from established disease (Tier C); none of these complications has predictive value.

**Table 4:** Development and validation characteristics of retinal-image-based prediction models for preeclampsia, organised according to TRIPOD + AI and PROBAST + AI items.

Item	Zhou et al. (2024) [98]	Wu et al. (2025), PROMPT [99]
Design; setting; period	Prospective cohort; Shanghai First Maternity and Infant Hospital, Tongji University; singleton pregnancies enrolled in 2020–2022	Prospective cohort; First Affiliated Hospital, Sun Yat-sen University; August 2021 to November 2023
Participants analysed	1138 (of 1209; 12 intrauterine deaths and 59 multiple pregnancies excluded)	1812 (of 2114 enrolled; 302 excluded)
Outcome events; definition	66 PE (92 HDP); ACOG Practice Bulletin No. 222	86 PE (4.7%), of which 29 were preterm; criteria given in the original Supplementary Material
Imaging	Non-mydriatic fundus camera at the first antenatal visit (<20 wks); automated quality filtering; eyes analysed not stated	Colour fundus photography (RetiCam3100) at 11–13^+6^ wks; right eye by default (left if image quality poor); automated quality assessment
Retinal predictor	Commercial deep learning hypertension detection model (Inception-ResNet-v2) developed in a general population, not in pregnancy; continuous “fundus score”	Deep learning vessel segmentation; five features retained by LASSO (arteriovenous ratio, vessel skeleton density, tortuosity density, angle-based tortuosity and its standard deviation)
Other predictors; model	Maternal risk factors; model specification and inclusion of blood pressure not reported	Guideline-based maternal risk factors and MAP; logistic regression
Development and internal validation	Combined model apparently developed and evaluated on the same data; no resampling or split reported (sensitivity analyses addressed the association only)	Ten-fold cross-validation (up-sampling applied only to the comparator machine learning algorithms, not to the logistic regression model); no hold-out test set; final model refitted on all data
Data-splitting level; leakage safeguards	Not applicable (no split reported); image model trained externally in a general population	One image per woman, so folds were participant-level; LASSO/stepwise feature selection performed before, not within, cross-validation (potential leakage)
External validation	None	None
Calibration	Not reported	Not reported
Discrimination for PE (AUC; 95% CI)	Fundus score: 0.845 (0.791–0.899); risk factors: 0.742 (0.675–0.810); combined: 0.883 (0.834–0.932), sensitivity: 0.72, and specificity: 0.93; fundus score aOR: 2.58 (1.88–3.62)	Baseline: 0.69 (0.63–0.76); retinal features: 0.74 (0.69–0.79); MAP model: 0.82 (0.78–0.87); PROMPT: 0.87 (0.83–0.90); preterm PE: 0.91 (0.85–0.97)
Comparison with established screening	None; FMF algorithm, MAP, UtA-PI and PlGF not evaluated in the same women	Incremental value over baseline and MAP models (DeLong test, NRI, and IDI); no FMF, UtA-PI or PlGF comparison
Key limitations; overall concern (PROBAST)	Image algorithm not designed for pregnancy; 66 events; apparent performance; adjustment set not reported; no calibration or external validation; high	Eighty-six events (29 preterm); non-nested feature selection; single centre and camera; no calibration or external validation; high

ACOG, American College of Obstetricians and Gynecologists; aOR, adjusted odds ratio; AUC, area under the receiver operating characteristic curve; CI, confidence interval; FMF, Fetal Medicine Foundation; HDP, hypertensive disorder of pregnancy; IDI, integrated discrimination improvement; LASSO, least absolute shrinkage and selection operator; MAP, mean arterial pressure; NRI, net reclassification improvement; PE, preeclampsia; PlGF, placental growth factor; PROBAST, Prediction model Risk of Bias Assessment Tool; PROMPT, the model designation used by Wu et al.; TRIPOD, Transparent Reporting of A Multivariable Prediction model for Individual Prognosis or Diagnosis; UtA-PI, uterine artery pulsatility index; wk, week. Performance estimates are not comparable between the two studies, which differ in population, timing, predictors, outcome prevalence and validation strategy.

**Table 5:** Intended uses of established clinical comparators and their implications for the evaluation of retinal markers.

Tool	Intended Use	Population and Timing	Key Performance Evidence	Evidence of Clinical Benefit	Implication for Retinal Markers
FMF competing-risks combined test [15,16,17,18,19,20]	Population screening to select women for aspirin prophylaxis	All pregnant women at 11–13^+6^ wks; maternal factors, MAP, UtA-PI and PlGF	Detects approximately three-quarters of preterm PE at a 10% screen-positive rate [15,16,17,18,19]	Aspirin in screen-positive women reduced preterm PE by about 60% [12,14]	Benchmark for first-trimester retinal models; requires comparison in the same cohort and demonstration of incremental value
sFlt-1/PlGF ratio [49,50]	Short-term prediction and diagnostic support in suspected PE	Women with suspected PE from the late second trimester (24^+0^–36^+6^ wks in PROGNOSIS)	Ratio ≤38 rules out PE within 1 week (negative predictive value: 99.3%) [49]	Accuracy evidence; supports triage and surveillance decisions	Not a first-trimester screening comparator; relevant to retinal markers proposed for diagnosis or triage (Tiers B–C)
PlGF-based testing [110]	Triage of suspected PE	Women with suspected PE at 20^+0^–36^+6^ wks	Revealed results shortened the time to clinical confirmation of PE [110]	Reduced time to diagnosis and severe maternal adverse outcomes in a cluster-randomised trial [110]	As above; retinal markers would need to add information in women already being tested
Uterine artery Doppler [112,113]	Component of combined screening; second-trimester risk assessment	First and second trimesters	Second-trimester indices predict PE, particularly early-onset disease, more accurately than first-trimester indices [113]	Benefit realised through combined screening and aspirin	Retinal markers should be evaluated in combination with, not instead of, Doppler indices
Ophthalmic artery Doppler [32,52]	Investigational adjunct to screening; differentiation of PE from other hypertensive disorders	Screening populations; women with hypertension in pregnancy	Pooled AUC of 0.71 → 0.79 when added to maternal factors [32]; independent association with PE among hypertensive women [52]	None demonstrated	Closest ocular analogue; overlapping CIs illustrate the difficulty of proving incremental value
fullPIERS model [111]	Prognosis of adverse maternal outcomes	Women with established PE	AUC of 0.88 (95% CI: 0.84–0.92) for adverse outcomes within 48 h in the development cohort [111]	Risk stratification of established disease	Benchmark for retinal markers proposed for severity assessment (Tier C)
Retinal deep learning models [98,99]	Not yet defined; candidate first-trimester risk stratification	11–20 wks	AUC of 0.87–0.88 for PE in development cohorts (Table 4)	None	Intended use, risk threshold and clinical action must be specified before validation and impact studies

AUC, area under the receiver operating characteristic curve; CI, confidence interval; FMF, Fetal Medicine Foundation; MAP, mean arterial pressure; PE, preeclampsia; PIERS, Pre-Eclampsia Integrated Estimate of Risk; PlGF, placental growth factor; sFlt-1, soluble fms-like tyrosine kinase-1; UtA-PI, uterine artery pulsatility index; wk, week. Performance figures for different tools derive from different populations and outcomes and should not be compared directly.

**Table 6:** Comparative appraisal of retinal assessment modalities for preeclampsia prediction and assessment.

Modality	Key Parameters	Principal Strengths	Principal Limitations	Evidence Maturity (Predominant Tier)
Qualitative fundoscopy [57,70]	Hypertensive retinopathy grade (arteriolar narrowing, haemorrhages, and exudates)	Inexpensive; universally available; severity marker	Subjective; observer-dependent; abnormal mainly in established/severe disease	Long-established; little predictive value (Tiers B–C)
Quantitative retinal vessel analysis (QRVA) [37,58,61,63,66,74,76,77]	CRAE, CRVE, AVR, fractal dimension, and tortuosity; flicker response	Objective and reproducible; standard fundus photograph; longitudinal cohort data	Modest effect sizes; overlapping distributions; confounding by blood pressure; non-standardised software/grading	Moderate; one longitudinal pre-diagnostic cohort (Tier A); otherwise, Tiers B and D
OCT/OCT angiography [31,36,38,54,55,60,68,69,79]	Choroidal thickness and flow; SCP/DCP vessel density; FAZ; RNFL/GCL	Depth-resolved; non-invasive microvascular detail; choroidal signal mechanistically plausible	Costly hardware; small, mostly post-diagnostic studies; inconsistent direction of choroidal and inner-retinal findings	Low–moderate; almost entirely Tiers B–C; one small longitudinal study from the first trimester
AI-assisted fundus photograph analysis [33,62,98,99]	Learned vascular features; end-to-end risk score	Low-cost, rapid, and operator-light; scalable; compatible with portable cameras	“Black-box” models; no external validation or calibration; device and dataset dependence	Emerging; two single-centre development studies (Tier A)

AI, artificial intelligence; AVR, arteriovenous ratio; CRAE, central retinal arteriolar equivalent; CRVE, central retinal venular equivalent; DCP, deep capillary plexus; FAZ, foveal avascular zone; GCL, ganglion cell layer; OCT, optical coherence tomography; RNFL, retinal nerve fibre layer; SCP, superficial capillary plexus.

**Table 7:** Study-level methodological appraisal of the key studies: assessment tool, principal issues and their likely impact on the results.

Study	Tier	Tool	Principal Methodological Issues	Likely Impact on Results	Overall Concern
Lupton et al. (2013) [63]	Ref.	NOS	Small sample (n = 53) of healthy women	Defines the physiological trajectory with limited precision	Moderate
Kiziltunc et al. (2020) [60]	Ref.	NOS	Cross-sectional pregnant vs. non-pregnant comparison; device-specific metrics	Shows that pregnancy alters OCTA metrics; absolute values not transferable	Moderate
Lupton et al. (2013) [66]	A	NOS	Small single-centre cohort; PE group older and heavier (adjusted); repeated comparisons; predictive accuracy not established	Supports temporality; effect sizes imprecise; predictive performance unknown	Moderate
Li et al. (2024) [77]	A	NOS	Outcome foetal growth rather than PE; hospital-based cohort	Indirect evidence only; not transferable to PE prediction	Moderate
Pehlivan et al. (2026) [38]	A, B	NOS	Small groups; predictive accuracy not established; single study	Hypothesis-generating; group differences may be imprecise	Moderate
Zhou et al. (2024) [98]	A	PROBAST (+AI)	Predictor algorithm not developed for pregnancy; 66 events; combined model evaluated in derivation data; adjustment set not reported; no calibration; no external validation	Optimistic (apparent) discrimination; transportability unknown	High
Wu et al. (2025) [99]	A	PROBAST (+AI)	86 events (29 preterm); feature selection outside cross-validation; single centre and camera; no calibration; no external validation	Optimistic cross-validated discrimination; transportability unknown	High
Phillipos et al. (2023) [37]	B	NOS (cross-sectional)	PE not separated from gestational hypertension; unadjusted comparison; small control group; single BP reading	Likely overstatement of a PE-specific effect (confounding by BP)	High
Bruckmann et al. (2015) [61]	B, D	NOS	Small groups; established disease; matched controls; adjusted for MAP	Robust to BP confounding but uninformative for prediction	Moderate
Lau et al. (2022) [52]	B	QUADAS-2	Spectrum limited to women referred with hypertension; adjusted for MAP and treatment	Applicable to differential diagnosis rather than screening	Moderate
Atas et al. (2014) [54]	B	NOS	Small; no adjustment for BP, gestational age or axial length described	Differences may reflect confounding; direction conflicts with other studies	High
Ciloglu et al. (2019) [78]	B	NOS	Multiple sectoral comparisons; no adjustment described	Risk of false-positive findings; limited comparability	High
Erkan Pota et al. (2024) [55]	B	NOS	Small groups; many parameters; no adjustment for BP described	Inconsistent parameter-level findings; possible confounding	High
Fayed et al. (2023) [36]	B	NOS	Very small groups; hypertension subtype unspecified	Imprecise, although the hypertensive comparator supports PE specificity	Moderate
Garg et al. (2014) [68]	B	NOS	Small; severe PE only; imaging in the early puerperium	Postpartum fluid shifts and treatment may influence thickness	Moderate
Silverman et al. (2023) [79]	B	NOS	Small; imaging within 72 h of delivery; mixed hypertensive group	Low power; timing limits inference about pregnancy	Moderate
Lee et al. (2021) [69]	C	NOS (cross-sectional)	Retrospective; no controls; gestational age uncontrolled; regression covariates unspecified	Association with angiogenic imbalance uncertain	High
Benschop et al. (2017) [76]	D	NOS	No pre-pregnancy retinal data; imaging missing in one-third; small PE group	Cannot separate pre-existing from pregnancy-related change; possible selection bias	Moderate
Sapantzoglou et al. (2025) [32]	A	AMSTAR 2	Six primary studies; overlapping confidence intervals	Added value plausible but uncertain	Moderate
Kitmiridou et al. (2026) [31]	B	AMSTAR 2	Heterogeneity precluded meta-analysis; primary studies at moderate or serious risk of bias	Synthesis appropriately inconclusive	Low–moderate
Liu et al. (2026) [33]	A	AMSTAR 2	Extreme heterogeneity (I^2^ > 99%); pooled discrimination dominated by development-phase estimates; inconsistent reporting of risk-of-bias judgements	Pooled AUC not transferable to clinical performance	Moderate

AMSTAR 2, A Measurement Tool to Assess Systematic Reviews 2; AUC, area under the receiver operating characteristic curve; BP, blood pressure; MAP, mean arterial pressure; ML, machine learning; NOS, Newcastle–Ottawa Scale; OCTA, optical coherence tomography angiography; PE, preeclampsia; PROBAST (+AI), Prediction Model Risk of Bias Assessment Tool, interpreted with its artificial intelligence extension; QUADAS-2, Quality Assessment of Diagnostic Accuracy Studies 2. Tiers are defined in Figure S2 (Ref., healthy-pregnancy reference study). Judgements are domain-based and reflect the information available in the published reports; they were not converted into numerical scores. Overall concern: low, moderate or high risk that the reported results misrepresent the true association or performance.

## Data Availability

No new data were created or analysed in this study. Data sharing is not applicable to this article.

## Supplementary Materials

The following supporting information can be downloaded at https://www.mdpi.com/article/10.3390/diagnostics16213676/s1. Section S1: Review methodology; Table S1: Search strategy and eligibility criteria; Figure S1: PRISMA-style flow diagram of study identification, screening and inclusion, including the studies added during revision; Figure S2: Classification of the evidence by the timing of retinal assessment relative to the clinical diagnosis of preeclampsia; Figure S3: Mechanism-to-evidence synthesis linking the systemic pathophysiology of preeclampsia to retinal findings by imaging modality. References [34,35,36,37,38,39,40,41,42,43] are cited in the Supplementary Materials.
