# Supplementary material for: Retinal Vasculature as a Non-Invasive Window for Preeclampsia Prediction and Assessment: From Fundoscopy to Artificial Intelligence-Assisted Retinal Imaging
Source: Diagnostics (Basel). 2026 Nov 6;16(21):3676. doi: 10.3390/diagnostics16213676 (PMC13763179; doi:10.3390/diagnostics16213676)
Supplement: Supplementary file 1 [file diagnostics-16-03676-s001.zip › diagnostics-4685310-supplementary.pdf]

Supplementary Materials

Retinal Vasculature as a Non-Invasive Window for Preeclampsia Prediction and Assessment: From Fundoscopy to Artificial Intelligence-Assisted Retinal Imaging

Dimitris Baroutis, Maria Fanaki, Konstantinos Koukoumpanis, Ioannis Fragiskos, Nikolaos Sindos, Michael Sindos, Vasilios Pergialiotis and George Daskalakis

This file contains the review methodology (Section S1); the complete search strategy and eligibility criteria (Table S1); the flow diagram of study identification, screening and inclusion (Figure S1); the classification of the evidence by the timing of retinal assessment relative to diagnosis (Figure S2); and a mechanism-to-evidence synthesis of the findings (Figure S3). Reference numbers refer to the reference list in the main text.

S1. Review Methodology

S1.1. Design and Literature Search

This work is a narrative review with a structured and reproducible literature search. A systematic review with meta-analysis was not undertaken because the objectives were interpretive rather than quantitative. The aim was to integrate heterogeneous modalities, study designs and outcomes within a common pathophysiological framework, not to estimate pooled effects, which the heterogeneity of devices, metrics and gestational windows would, in any case, preclude. To limit the selection and interpretation biases inherent in narrative reviews, the search, eligibility criteria, evidence classification and appraisal approach were defined explicitly and are reported here. The Preferred Reporting Items for Systematic Reviews and Meta-Analyses (PRISMA) 2020 statement was used as a template for reporting the identification and selection of studies [34], without any claim to a systematic review methodology. The reporting also addresses the quality criteria of the Scale for the Assessment of Narrative Review Articles (SANRA) [35].

PubMed/MEDLINE, Scopus, Web of Science and the Cochrane Library were searched from database inception to 30 June 2026 (date of the last search). The search combined terms for the hypertensive disorders of pregnancy (preeclampsia, pre-eclampsia, eclampsia, gestational hypertension, and hypertensive disorders of pregnancy) with terms for retinal and ocular vascular assessment. The latter comprised retina, retinal vessel, retinal vascular calibre, fundoscopy, fundus photography, optical coherence tomography, OCT angiography, ophthalmic artery Doppler, deep learning, artificial intelligence and oculomics. The complete Boolean search string is reported in Table S1. The reference lists of retrieved articles and of relevant reviews were screened to identify additional sources.

S1.2. Eligibility Criteria and Study Selection

Eligible sources included original studies in pregnant or postpartum women, or in women with a history of preeclampsia, that evaluated retinal or ocular measures in relation to preeclampsia or the hypertensive disorders of pregnancy. Eligible designs included prospective or retrospective cohort, case–control, cross-sectional, diagnostic accuracy and prediction model studies. Eligible measures included retinal or choroidal structure, retinal vascular calibre or geometry, retinal vascular function, ophthalmic or central retinal artery Doppler indices, and artificial intelligence-derived retinal features. Systematic reviews and meta-analyses of these topics were also eligible. Case reports and case series were considered only for the severe ocular complications described in Section 7 of the main text. Only English-language full-text publications were included; conference abstracts and studies without a retinal or ophthalmic vascular measure were excluded. The literature on the pathophysiology of preeclampsia, established screening algorithms, retinal vascular epidemiology and non-obstetric retinal artificial intelligence was cited to provide context and methodological benchmarks but did not form part of the appraised evidence base.

Records were screened by title and abstract and then in full text against these criteria; the flow of records is shown in Figure S1. The search yielded 2092 records (2074 from the databases and 18 from reference lists), of

which 38 studies were included. Three further eligible studies, by Fayed et al. [36], Phillipos et al. [37] and Pehlivan et al. [38], were identified during the revision of this manuscript and added, giving a total of 41 included studies. The most informative of the included studies are tabulated in Sections 5, 6 and 8 of the main text.

S1.3. Evidence Classification, Quality Appraisal and Synthesis

The predictive value of a marker can be established only when it is measured before the outcome occurs. Each included study was, therefore, classified according to the timing of retinal assessment relative to the clinical diagnosis of preeclampsia (Figure S2). Tier A (early prediction) comprises assessment before diagnosis, either preconception or in the first or early second trimester, in cohorts followed to delivery. Tier B (diagnostic discrimination) comprises assessment at or after diagnosis, comparing women with preeclampsia with normotensive or other hypertensive pregnancies. Tier C (severity and prognostic assessment) comprises studies relating retinal findings to disease severity, angiogenic markers or complications within established preeclampsia. Tier D comprises postpartum and long-term follow-up. Studies of healthy pregnancy, which define the physiological reference against which pathological change must be judged, were considered separately. For each key study, we extracted the design and setting, sample size and number of preeclampsia cases, gestational age at imaging, and the definition and subtype of preeclampsia where reported. We also extracted the imaging device and metrics, the principal quantitative results and the confounders adjusted for. For prediction model studies, we additionally extracted the development and validation samples, event counts, validation strategy and level of data splitting, calibration, and measures taken against data leakage.

Methodological quality was appraised with instruments matched to study design. The Newcastle–Ottawa Scale was used for cohort, case–control and cross-sectional studies [39], and the revised Quality Assessment of Diagnostic Accuracy Studies tool (QUADAS-2) for diagnostic accuracy studies [40]. Prediction model studies were assessed with the Prediction Model Risk of Bias Assessment Tool (PROBAST) [41], interpreted in light of its extension for artificial intelligence models (PROBAST+AI) [42]. Systematic reviews were assessed with A Measurement Tool to Assess Systematic Reviews (AMSTAR 2) [43]. Consistent with the narrative design, these instruments were used to structure domain-level judgements rather than to generate numerical quality scores. Judgements were summarised as low, moderate or high concern, together with the principal methodological issues and their likely impact on the results. No study was excluded on the basis of quality, and the study-level judgements are presented in Section 10.1 of the main text. Findings were synthesised narratively, organised by modality and evidence tier. Greater weight was given to prospective, pre-diagnostic, adequately adjusted and externally validated evidence than to cross-sectional comparisons in established disease, and the direction, magnitude and consistency of associations were considered alongside their statistical significance. No meta-analysis was undertaken, because devices, metrics, comparison groups and gestational windows differed too widely for pooled estimates to be meaningful. The use of generative artificial intelligence in the preparation of this manuscript is described in the Acknowledgments of the main text.

Table S1. Search strategy and eligibility criteria.

Item	Specification
**Review design**	Narrative review with a structured and reproducible literature search; PRISMA 2020 used as a reporting template for study identification and selection only; no meta-analysis.
**Databases**	PubMed/MEDLINE, Scopus, Web of Science and the Cochrane Library.
**Period searched; date of last search**	From database inception to 30 June 2026; last search performed on 30 June 2026.
**Search string**	(preeclampsia OR pre-eclampsia OR eclampsia OR "gestational hypertension" OR "hypertensive disorders of pregnancy") AND (retina OR "retinal vessel" OR "retinal vascular calibre" OR fundoscopy OR "fundus photography" OR "optical coherence

Item	Specification
	tomography" OR "OCT angiography" OR "ophthalmic artery Doppler" OR "deep learning" OR "artificial intelligence" OR oculomics).
**Additional sources**	Reference lists of retrieved articles and of relevant reviews; three further eligible studies identified during the revision of this manuscript (Fayed 2023; Phillipos 2023; Pehlivan 2026).
**Language**	English-language full-text publications.
**Eligible designs**	Prospective or retrospective cohort, case–control, cross-sectional, diagnostic accuracy and prediction model studies; systematic reviews and meta-analyses. Case reports and case series only for severe ocular complications.
**Population**	Pregnant or postpartum women, or women with a history of preeclampsia; studies of healthy pregnancy as physiological reference.
**Index measures**	Retinal or choroidal structure; retinal vascular calibre or geometry; retinal vascular function; ophthalmic or central retinal artery Doppler indices; and artificial intelligence-derived retinal features.
**Outcome/condition**	Preeclampsia or the hypertensive disorders of pregnancy (definitions as reported by the original studies).
**Exclusions**	No retinal or ophthalmic vascular measure; non-pregnant populations (other than reference or long-term follow-up studies); conference abstracts; and narrative reviews and editorials (used only for reference-list screening and context).
**Evidence classification**	Tier A, imaging before diagnosis (early prediction); Tier B, at or after diagnosis (diagnostic discrimination); Tier C, within established disease (severity and prognosis); and Tier D, postpartum and long-term follow-up. Healthy pregnancy reference studies considered separately.
**Data extracted**	Design and setting; sample size and number of preeclampsia cases; gestational age at imaging; definition and subtype of preeclampsia where reported; device and metrics; principal quantitative results; confounders adjusted for; and for prediction models, development and validation samples, event counts, validation strategy, data-splitting level, calibration and leakage safeguards.
**Quality appraisal**	Newcastle–Ottawa Scale (cohort, case–control and cross-sectional studies); QUADAS-2 (diagnostic accuracy studies); PROBAST interpreted with PROBAST+AI (prediction models); and AMSTAR 2 (systematic reviews). Domain-level judgements summarised as low, moderate or high concern; no numerical scores; no exclusion on quality.
**Synthesis**	Narrative synthesis organised by modality and evidence tier; greater weight given to prospective, pre-diagnostic, adequately adjusted and externally validated evidence; direction, magnitude and consistency of associations considered alongside statistical significance.

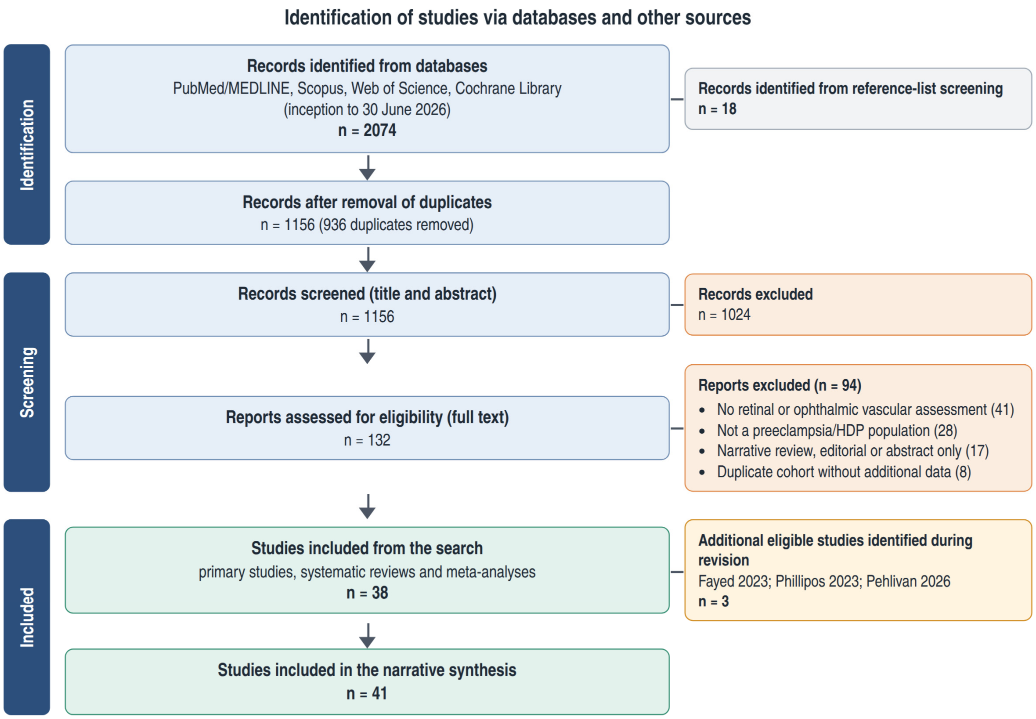

Flow diagram adapted from the PRISMA 2020 statement and used as a reporting template for this narrative review. HDP, hypertensive disorders of pregnancy.

Figure S1. PRISMA-style flow diagram of study identification, screening and inclusion. Of 2092 records identified (2074 across four databases and 18 through reference-list screening; database inception to 30 June 2026), 38 studies were included from the search. Three further eligible studies identified during the revision of this manuscript were added, giving 41 studies included in the narrative synthesis. The PRISMA 2020 statement was used as a reporting template only.

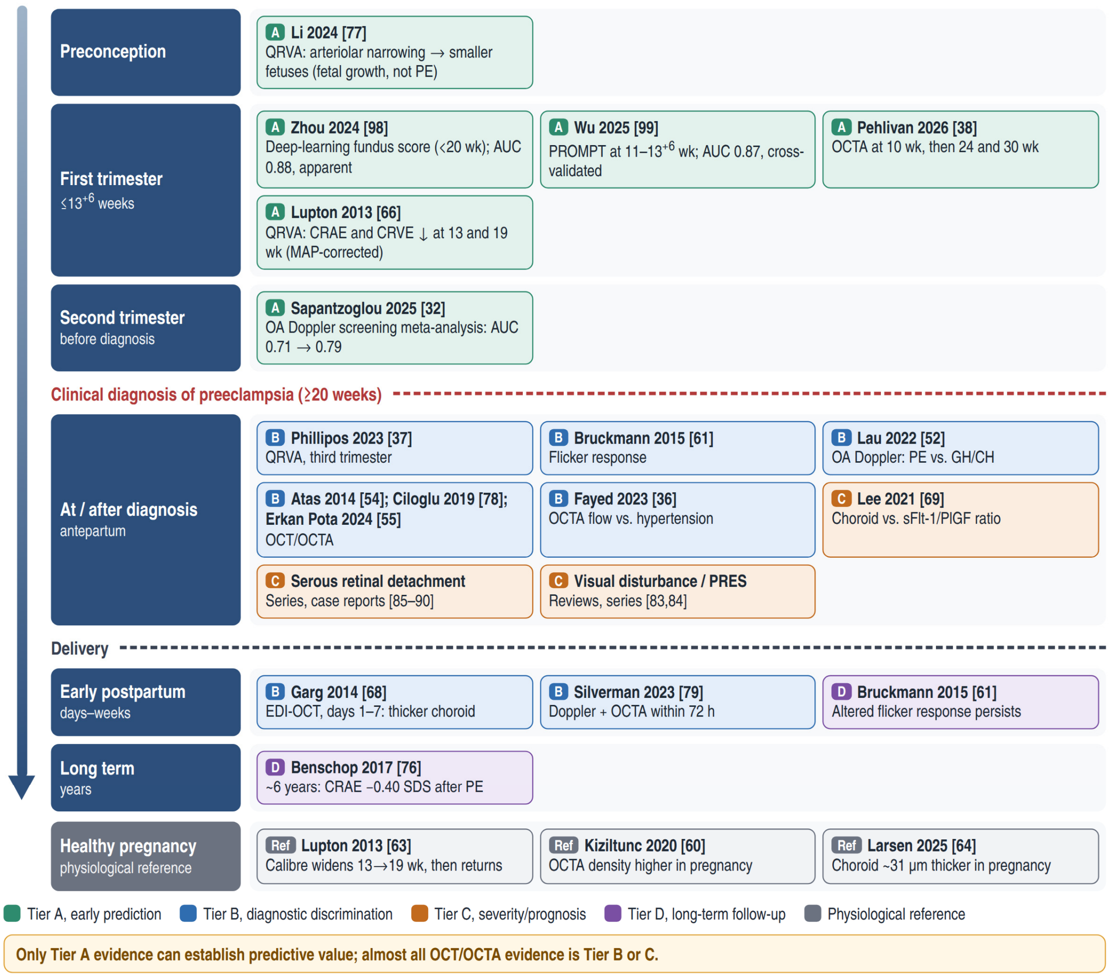

Figure S2. Classification of the evidence by the timing of retinal assessment relative to the clinical diagnosis of preeclampsia. Tier A (early prediction) comprises assessment before diagnosis (preconception or in the first or early second trimester) in cohorts followed to delivery. Tier B (diagnostic discrimination) comprises assessment at or after diagnosis; Tier C (severity and prognostic assessment), studies within established disease; and Tier D, postpartum and long-term follow-up. Representative studies are positioned according to the timing of imaging; studies of healthy pregnancy that define the physiological reference are shown separately. Only Tier A evidence can establish predictive value. *AI, artificial intelligence; CRAE, central retinal arteriolar equivalent; CRVE, central retinal venular equivalent; DL, deep learning; OA, ophthalmic artery; OCT, optical coherence tomography; OCTA, OCT angiography; PE, preeclampsia; QRVA, quantitative retinal vessel analysis; sFlt-1/PlGF, soluble fms-like tyrosine kinase-1-to-placental growth factor ratio.*

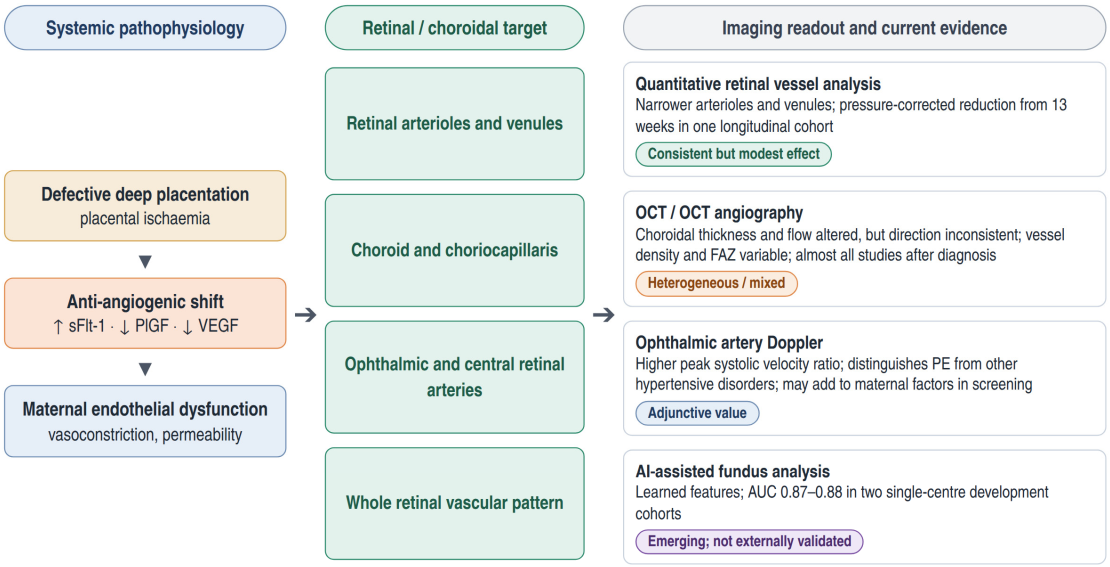

AUC, area under the receiver-operating-characteristic curve; FAZ, foveal avascular zone; OCT, optical coherence tomography; PE, preeclampsia; PlGF, placental growth factor; sFlt-1, soluble fms-like tyrosine kinase-1; VEGF, vascular endothelial growth factor.

Figure S3. Mechanism-to-evidence synthesis linking the systemic pathophysiology of preeclampsia to retinal findings by imaging modality, annotated with the direction and consistency of the reported associations. The evidence for optical coherence tomography is derived almost entirely from women imaged after diagnosis, and the artificial intelligence models have not been externally validated. Sections 5–8 and Tables 1, 2 and 4 of the main text provide study-level detail.
